# Blocking the MIF-CD74 axis augments radiotherapy efficacy for brain metastasis in NSCLC via synergistically promoting microglia M1 polarization

**DOI:** 10.1186/s13046-024-03024-9

**Published:** 2024-04-29

**Authors:** Lichao Liu, Jian Wang, Ying Wang, Lingjuan Chen, Ling Peng, Yawen Bin, Peng Ding, Ruiguang Zhang, Fan Tong, Xiaorong Dong

**Affiliations:** 1 Cancer Center, Union Hospital, Tongji Medical College, Huazhong University of Science and Technology, Wuhan, Hubei 430022 China; 2 Hubei Key Laboratory of Precision Radiation Oncology, Wuhan, Hubei 430022 China; 3 Institute of Radiation Oncology, Union Hospital, Tongji Medical College, Huazhong University of Science and Technology, Wuhan, Hubei 430022 China

**Keywords:** NSCLC, Brain metastases, Microglia, MIF, CD74, Radiotherapy

## Abstract

**Background:**

Brain metastasis is one of the main causes of recurrence and death in non-small cell lung cancer (NSCLC). Although radiotherapy is the main local therapy for brain metastasis, it is inevitable that some cancer cells become resistant to radiation. Microglia, as macrophages colonized in the brain, play an important role in the tumor microenvironment. Radiotherapy could activate microglia to polarize into both the M1 and M2 phenotypes. Therefore, searching for crosstalk molecules within the microenvironment that can specifically regulate the polarization of microglia is a potential strategy for improving radiation resistance.

**Methods:**

We used databases to detect the expression of MIF in NSCLC and its relationship with prognosis. We analyzed the effects of targeted blockade of the MIF/CD74 axis on the polarization and function of microglia during radiotherapy using flow cytometry. The mouse model of brain metastasis was used to assess the effect of targeted blockade of MIF/CD74 axis on the growth of brain metastasis.

**Result:**

Our findings reveals that the macrophage migration inhibitory factor (MIF) was highly expressed in NSCLC and is associated with the prognosis of NSCLC. Mechanistically, we demonstrated CD74 inhibition reversed radiation-induced AKT phosphorylation in microglia and promoted the M1 polarization in combination of radiation. Additionally, blocking the MIF-CD74 interaction between NSCLC and microglia promoted microglia M1 polarization. Furthermore, radiation improved tumor hypoxia to decrease HIF-1α dependent MIF secretion by NSCLC. MIF inhibition enhanced radiosensitivity for brain metastasis via synergistically promoting microglia M1 polarization in vivo.

**Conclusions:**

Our study revealed that targeting the MIF-CD74 axis promoted microglia M1 polarization and synergized with radiotherapy for brain metastasis in NSCLC.

**Supplementary Information:**

The online version contains supplementary material available at 10.1186/s13046-024-03024-9.

## Background

Lung cancer is one of the most common cancers and a prime cause of cancer-related deaths worldwide [1]. Brain metastases are present in 57% of lung cancer patients, with 20% of patients presenting with brain metastasis (BM) at initial diagnosis and 50% presenting at relapse [2]. Treatment options for brain metastasis include whole-brain radiotherapy (WBRT) or stereotactic radiotherapy (SRT) alone or in combination with surgical resection [3]. However, the treatment was not always effective.

The tumor microenvironment (TME) plays a crucial role in the outcome of radiotherapy for brain metastasis [4–6]. Tumor-associated macrophages (TAMs), including microglia, are the most common cells in the TME of brain metastasis and have key homeostatic functions that influence tumor maintenance and growth [7–9]. Depending on environmental cues, they can be polarized into two distinct phenotypes: the pro-inflammatory M1 phenotype and the anti-inflammatory M2 phenotype [10]. M1 macrophages are activated in response to lipopolysaccharides and IFN-γ [11, 12], whereas the M2 phenotype can be induced with IL-4/IL-13, IL-10, TGF-β, and macrophage colony-stimulating factor (M-CSF) [13–15]. The majority of TAMs in the TME demonstrate an M2-like phenotype and promote tumor growth and metastasis [16–19]. The increased presence of TAMs is correlated with poorer clinical outcomes in different types of cancers [20–23]. Experimental data have shown that radiation recruits both M1 and M2 macrophages in murine tumor models of tumors [24, 25]. The balance of M1 versus M2 macrophages produced following radiation may depend on the radiation dose [25–29]. Pre-existing macrophages and the subsequently recruited following radiation play an important role in both the initial anti-tumor immune response and the later formation of the immunosuppressive pro-tumoral microenvironment [30–32]. Radiotherapy resistance may be attributed in part to the “pro-tumor” M2-like phenotype of macrophages following radiation [33, 34]. Targeting macrophage infiltration and polarization in combination with radiation has demonstrated tremendous synergy in preclinical models and early phase clinical trials [33, 35–40].

Elevated expression of the macrophage migration inhibitory factor (MIF) has been reported in various solid tumors including colon cancer, lung cancer, breast cancer, pancreatic cancer, melanoma, nasopharyngeal carcinoma, cervical adenocarcinoma, and prostate cancer, and correlated with poor prognosis in patients with cancer [41]. MIF exerts its pro-tumor effects by modulating the immunosuppressive TME, mainly via regulating the CD74 signaling in macrophages and dendritic cells (DCs) [42–44]. CD74, a type-II transmembrane glycoprotein on the cell surface, acts as a chaperone and a transport cofactor for MHC II, responsible for appropriate antigen presentation and a receptor for MIF [45]. The MIF-CD74 axis has been shown to play pivotal roles in both activating an oncogenic signaling pathway [46] and establishing the immunosuppressive microenvironment [47] to promoting tumor growth. In primary brain tumors, MIF has been found to maintain tumorigenicity by directly inhibiting p53, and also is considered an adverse prognostic factor [48, 49]. Research on the association between the BM of NSCLC and the MIF/CD74 axis is limited, with only a few studies discovering that MIF is a tumor cell-oriented factors involved in crosstalk between tumor cells and astrocytes and is associated with tumor angiogenesis [50, 51]. In melanoma, Fukuda, Y. et al. showed that inhibiting the MIF/CD74/AKT signaling pathway suppressed melanoma proliferation by inducing apoptosis [44]. Treatment with rSmeg-hMIF-hIL-7, a vaccine which could induce anti-MIF immune response, enhanced activation of CD4 + and CD8 + T cells in a MC38 tumor-bearing mouse model [52]. Therefore, inhibiting MIF-CD74 signaling on macrophages via anti-MIF antibodies or MIF inhibitors could restore the antitumor immune response in tumor microenvironments.

Hypoxia, a hallmark of cancer, is a potent inducer of MIF [53]. MIF is a HIF-1α-dependent gene and a key regulator of HIF-1α stabilization [54]. Radiation could ameliorate tumor hypoxia and reduces the transcription of HIF-1α [55]. In this study, we reported that radiotherapy ameliorated tumor hypoxia, downregulated HIF-1α expression and MIF secretion in NSCLC, and inhibited the binding of MIF to CD74 in microglia. The transformation of M2 microglial cells into M1 phenotype was regulated by the PI3K/AKT pathway, which enhanced radiation sensitivity. The HIF-1α/MIF/CD74 axis could serve as a target for overcoming radioresistance and treatment for brain metastases in NSCLC.

## Materials and methods

### Cell culture

Murine Lewis lung cancer cell (LLC) and human NSCLC cell H1975 (*EGFR* T790M, L858R) were purchased from American Type Culture Collection (ATCC). Mouse microglia BV2 was purchased from Procell Life Science & Technology CO.,Ltd (Wuhan, China). HCC827 (human lung adenocarcinoma cell line, *EGFR* exon 19 deletion) was acquired from the Institute of Biochemistry and Cell Biology of the Chinese Academy of Sciences(Shanghai, China). Beas-2B (human lung epithelial cell line) and PC-9 (human lung adenocarcinoma cell line, *EGFR* exon 19 deletion) were maintained in the laboratory of cancer center of Union Hospital, Tongji Medical College, Huazhong University of Science and Technology. BV2 and LLC cells were cultured in Dulbecco's Modified Eagle Medium (DMEM) supplemented with 10% fetal bovine serum (FBS) at 37 °C in a humidified incubator with 5% CO_2_. DMEM and FBS were purchased from Gibco (Carlsbad, USA). HCC827, H1975, Beas-2B and PC-9 cells were cultured in RPMI 1640 medium (Gibco) supplemented with 10% FBS. Cells were grown at 37 °C in a humidified atmosphere of 5% CO_2_, and cells at the logarithmic phase were used in the experiments. To mimic hypoxia tumor environment, cells were incubated in a hypoxic incubator to create a hypoxic microenvironment. KC7F2 (MCE, 40 μM) was used as a HIF-1 inhibitor. The exact concentration details have been indicated in the corresponding figure.

### ELISA

MIF wase quantified using Mouse MIF ELISA kit (cat. no. ab209885; Abcam) according to manufacturer's instructions.

### Radiation

Cells were placed 100 cm away from the radioactive source. Cells and animals were exposed to the indicated dose of radiation by 6MV X rays at 600MU/min (Varian, USA). BV-2 was collected for analysis after radiation at the indicated time.

### Knockdown of gene

Lentiviral CD74 sh-RNA, Lentiviral MIF sh-RNA and the negative control constructs, which carry the puromycin resistance gene, and the corresponding virus were purchased from Huazhong Agricultural University (Wuhan, China). The titer of lentivirus was determined via serial dilution. Then, 1 × 10^8^ TU/ml lentivirus and 2 μg/ml polybrene were used to transduce BV-2, which were seeded in 6-well plates. Then the treated-cells were incubated for 24 h. After the medium was refreshed, the cells were cultured for another 48 h. Stable cell lines were selected by using puromycin.

### Quantitative real-time polymerase chain reaction (RT-qPCR)

The mRNA levels of microglial M1/M2 markers (iNOS, CD86, YM-1 and Arg-1) were measured by qRT-PCR. Briefly, total RNA was extracted with Trizol reagent and complementary DNA synthesis was performed with TaKaRa cDNA synthesis kit (TaKaRa, Japan) according to the manufacturer's instructions. The total PCR system contained cDNA, SYBR Green DNA polymerase, RNase-free water, and primers. The primer sequences were designed by using the Beacon Designer software package (Bio-Rad). The primers are shown as follows: iNOS sense 5′- TTC TCA GCC CAA CAA TAC AAGA-3′ and anti-sense 5′-GTG GAC GGG TCG ATG TCAC-3′; Arg-1 sense 5′-CTC CAA GCC AAA GTC CTT AGAG-3′, and anti-sense 5′-GGA GCT GTC ATT AGG GAC ATCA-3′; GAPDH sense 5′-AGG TCG GTG TGA ACG GAT TTG -3′ and anti-sense 5′- GGG GTC GTT GAT GGC AACA -3′; CD86 sense 5′- TGT TTC CGT GGA GAC GCA AG -3′ and anti-sense 5′- TTG AGC CTT TGT AAA TGG GCA -3′; YM-1 sense 5′- CAG GTC TGG CAA TTC TTC TGAA -3′ and anti-sense 5′- GTC TTG CTC ATG TGT GTA AGT GA -3′; CD74 sense 5′- GAC GAG AAC GGC AAC TAT CTG -3′ and anti-sense 5′- GTT GGG GAA GAC ACA CCA GC-3′. The experiments were repeated three times.

### Western blot analysis

Total proteins were extracted from the indicated tissues and cells by using a protein extraction kit (Pierce Biotechnology Inc., IL, USA) in accordance with the manufacturer’s protocol. The collected proteins were loaded and separated on sodium dodecyl sulfate (SDS)-polyacrylamide gels, and then transferred onto polyvinylidene fluoride (PVDF) membranes which were rocked gently for at least 1 h in blocking buffer (5% milk in TBST). Subsequently, the membranes were incubated overnight with primary antibodies; namely, iNOS (1:1000, ab15323, Abcam, UK), Ym-1 (1:1000, ab192029, Abcam, UK), CD86 (1:1000, ab167720, Abcam, UK), Arg-1 (1:1000, 89,007, Abcam, UK), MIF (1:1000, ab187064, Abcam, UK), CD74 (1:1000, ab270265, Abcam, UK), ERK1/2 (1:1000, ab184699, Abcam, UK), p-ERK1/2 (1:1000, ab278538, Abcam, UK), AKT (1:1000, ab8805, Abcam, UK), p-AKT (1:1000, ab38449, Abcam, UK),and GAPDH (1:2000, AC002, ABclonal, China). After incubating with horseradish peroxidase-labeled goat anti-rabbit or goat anti-mouse secondary antibody (1:5000, BA1054/BA1050, Boster, China) for 1 h, the protein bands were detected with the ECL Western blot detection kit in an enhanced chemiluminescence system.

### Phagocytosis assay

The BV2 cells were inoculated at a density of 50% in six-well plate, and then co-cultured with FITC-labeled latex beads (10 μl latex beads diluted in 2 ml DMEM) for 2 h. After washing with PBS for 3 times, the cells were fixed with 4% paraformaldehyde for 30 min, and then treated with 0.1% Triton X-100 for 3–5 min to increase cell permeability. Subsequently, the cells were incubated with Phalloidin-conjugate working solution for 20–90 min at room temperature. After wash process, the slides were sealed with anti-fluorescence quenching agent and observed under a fluorescence microscope. The phagocytic activiy of BV2 cells is measure by number of microspheres (green).

### Animal model of brain metastasis

Six-week-old female C57BL/6 J mice (Beijing Vital River Laboratory Animal Technology Co. Ltd, China) were housed under specific pathogen-free conditions in the same facility with the constant bred environment. The animal model of brain metastasis was established as described previously [56]. LLC cells labeled with luciferase (LLC-Luc, 4 × 10^5^ in 0.1 mL PBS) were slowly injected into the intracarotid artery of mice. Two weeks later, the growth of brain tumors was monitored by bioluminescence imaging in the IVIS Lumina imaging system (In Vivo FX PRO, Bruker Corporation) after injecting with D-luciferin intraperitoneally (150 mg/kg) in mice once a week. Neutral liposomal clodronate was used for macrophage depletion (F70101C-A-2, FormuMax), and injected intracerebroventricularly with a stereotaxic instrument at 2.0 mm posterior, 1.5 mm lateral, and 2.5 mm inferior to the level of fontanelle, 10 ul/mouse. ISO-1 was administered via intraperitoneal injection at a dose of 35 mg/kg, every two days, for a total of two weeks. All animal studies were conducted following the instructions of the Animal Care Committee of Tongji Medical College, Huazhong University of Science and Technology, China.

### Flow cytometry analysis

The tumor microenvironment of brain metastasis was analyzed by flow cytometry. The mouse brains were collected and kept with PBS on ice, then cut into small pieces and minced. Subsequently, the samples were digested for 1 h with 0.5 mg/mL collagenase type IV, DNase I 20 U/mL at 37 °C, then filtered and separated by Percoll (GE Healthcare Life Sciences) with density gradient (70%, 37%, 30%). To assay microglia cells in the animal model of brain metastasis [57], the single cells were stained with FITC-conjugated anti-CD11b antibodies (101,206, BioLegend, USA), Zombie NIR™ Fixable Viability Kit (423,106, BioLegend, USA), CD45 Brilliant Violet 510 (109,837, BioLegend, CA), CD86 PE (159,204, BioLegend, USA), and CD206 Brilliant Violet 421 (141,717, BioLegend, USA) for 20 min at 4℃. Microglia were identified with CD11b^+^CD45^low^. The M1 type microglia were labeled by CD86, and the M2 microglia were labeled by CD206. To assay T cells in the animal model of brain metastasis [58], The single cells were stained with PE anti-human FOXP3 Recombinant Antibody (364,703, BioLegend, USA), Zombie NIR™ Fixable Viability Kit (344,731, BioLegend, USA), Brilliant Violet 510™ anti-human CD8 Antibody (109,837, BioLegend, USA), Brilliant Violet 421™ anti-mouse IFN-γ Antibody (505,829, BioLegend, USA), PE/Cyanine7 anti-human CD45 Antibody (368,531, BioLegend, USA), FITC anti-human CD3 Antibody (317,305, BioLegend, USA), APC anti-mouse CD4 Antibody(100,411, BioLegend, USA).

### Immunofluorescence staining

The cells were fixed in 4% paraformaldehyde for 30 min at room temperature, and then blocked with 10% goat serum (GTX27481, Gene-Tex, USA) for 1 h at room temperature. The samples were incubated at 4 °C overnight with the following primary antibodies, such as iNOS (1:100, ab15323, Abcam, UK), CD86(1:100, ab239075, Abcam, UK), Arg-1(1:100, ab239731, Abcam, UK), CD34(1:100, ab81289, Abcam, UK), α-SMA (1:100, ab124964, Abcam, UK), IBA(1:100, ab178846, Abcam, UK) and F4/80 (1:50, ab6640, Abcam, UK). Subsequently, the sections were incubated with mouse anti-rabbit IgG (1:1000, #4408/4409, Cell Signaling Technology, USA), rabbit anti-mouse IgG (1:1000, #4412/4413, Cell Signaling Technology, USA), and donkey anti-rat IgG (1:1000, A21209, Invitrogen, USA) for 1 h at room temperature. After washing, the sections were mounted with a DAPI-containing antifade solution and observed under a microscope (BX63, Olympus) and a confocal microscope (Zeiss, LSM800).

### Cell viability assay

The cell viability was evaluated by the cell counting kit-8 assay (CCK-8, Dojindo Laboratories, Japan) according to the manufacturer’s protocols. Briefly, the cells were seeded in 96-well plates (5 × 10^3^cells per well) and treated as indicated. For detection, 10 μl of CCK-8 solution was added to the culture medium and incubated for 2 h in 5% CO_2_ at 37 °C. Then, the absorbance at 450 nm was measured. All experiments were repeated at least three times.

### Electrophoretic mobility shift assay (EMSA)

The MIF probe used in this assay was synthesized by Qingke Biotechnology Co., Ltd. The probe sequences are as follows: Forward: 5′-GTCAGGCACGTAGCTCAGC-3′; Reverse: 5′-GCTGAGCTACGTGCCTGAC-3′. The 5X binding buffer was made using 500 µl 1 M Tris (pH 7.5), 2.5 ml 1 M NaCl, 50 µL DTT, 100 µL 0.5 M EDTA (pH 8), 1.5 g Ficoll, and water to 10 ml. Reagents for the binding reaction were 4 µL 5X binding buffer, 1 µL Poly, 10.5 µL water, 1 µL MIF probe, and 1.5 µL rabbit anti-HIF-α antibodies (Cell Signaling Technology, Whitby ON, Canada). The mixtures were incubated at room temperature in the dark for 30 min to allow HIF-1α and MIF to bind. A 4% polyacrylamide gel was prepared containing 400 mM Tris at pH 7.5 and 25 mM EDTA. The unloaded gel was pre-run at constant 30 mA for 1 h at 4 °C and the loaded gel was run at 30 mA for 2 h at 4 °C. Add the prepared ECL mixture dropwise onto the protein side of the membrane and expose it in a darkroom.

### Patients’ samples

Non-small cell lung cancer (NSCLC) patients with BM were enrolled and BM tissues and their corresponding paracancerous tissue samples were collected in the Union Hospital of Tongji Medical College, Huazhong University of Science and Technology for the detection of Macrophage Migration Inhibitory Factor (MIF) protein level. This study was approved by the Institutional Review Board of Huazhong University of Science and Technology. Written informed consent was obtained from legal guardians of the patients.

### Immunohistochemical (IHC) staining

After dewaxing in xylene and rehydration in graded alcohols, the tissue sections were boiled in citrate buffer, pre-incubated with H_2_O_2_, and then blocked with rabbit or goat serum (DAKO, Denmark). Subsequently, the sections were incubated with a primary antibody and then with an HRP-conjugated secondary antibody. The proteins of interest were visualized by using diaminobenzidine before counterstaining with hematoxylin. The primary antibodies used were as following: MIF (ab7207, Abcam, USA), CD68 (ab283654, Abcam, USA) and HIF-α (ab51608, Abcam, USA).

### Analysis of and public cancer data

The UALCAN website (ualcan.path.uab.edu/index.html) was used to analyze TCGA database data. LUNG CANCER EXPLORER website was used to analyze survival data of NSCLC patients.

### Statistical analysis

Results were reported as the mean ± standard deviation (SD). The number of samples in each group is indicated in the legend of each figure. Unpaired two-tailed Student’s test was used to compare two independent groups. One-way analysis of variance (ANOVA) was used when three or more independent groups were compared. For survival analysis of BM mouse model, data were plotted and compared using the log-rank test or Gehan-Breslow-Wilcoxon test. A two-tailed test with *P* < 0.05 was significant. The analysis was performed with the GraphPad Prism Software version 7.. *represents *P* < 0.05, ** represents *P* < 0.01, *** represents *P* < 0.001, **** represents *P* < 0.0001, and ns represents not significant.

## Results

### MIF was elevated in NSCLC and indicated a poor prognosis

**Fig. 1:**
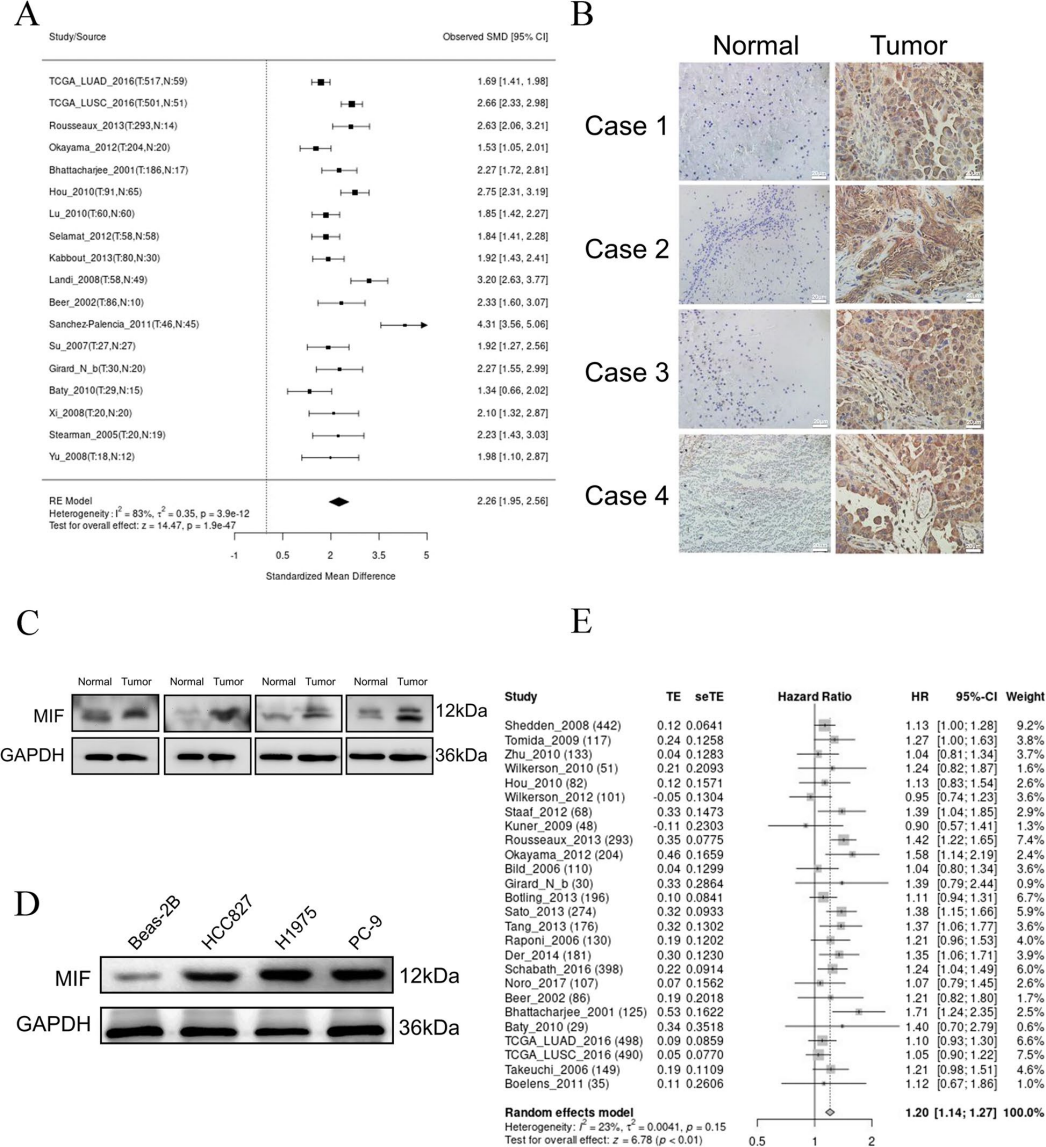
MIF was elevated in NSCLC and indicated a poor prognosis **a**. Meta analysis of MIF expression in tumor tissue and normal tissues in LUNG CANCER EXPLORER website. **b** IHC staining of MIF in NSCLC brain metastatic tissue and paired paracancerous tissue **c**. Difference of MIF protein level in NSCLC brain metastases and paired paracancerous surgical tissues. **d**. Difference of MIF protein level in Beas-2B, HCC827, H1975, and PC-9 cells. **e**. Survival meta-analysis of correlation between the expression of MIF and survival of NSCLC patient in LUNG CANCER EXPLORER website

To investigate the potential mechanism of brain metastasis (BM) in NSCLC, we found that *MIF* was upregulated in lung cancer compared to adjacent tissues by analyzing the public NSCLC transcriptomic data via the LUNG CANCER EXPLORER website (Fig. 1A). To confirm the upregulation of MIF expression in brain metastases of NSCLC patients, we collected several pairs of brain metastases and their paired adjacent tissues. Using immunohistochemistry and Western blot (4 pairs of paired tissues respectively), we found that MIF was upregulated in BM tumors compared with their adjacent tissues (Fig. 1B,C). We assessed the protein expression of MIF in three human-derived NSCLC cell lines harboring EGFR mutations and compared them with human lung epithelial cell line. As shown in Fig. 1D, MIF was significantly upregulated in NSCLC cell lines (HCC827, H1975, and PC-9) compared with the lung epithelial cell line (Beas-2B). Finally, we analyzed the LUNG CANCER EXPLORER database and found that the low expression of MIF is benefit for the survival of lung cancer (Fig. 1E). Taken together, these data demonstrated that expression of MIF was upregulated in NSCLC with BM, and high expression of MIF was associated with poor prognosis.

### Inhibiting CD74 in microglia in combination with radiation synergistically promoted M1 polarization

**Fig. 2:**
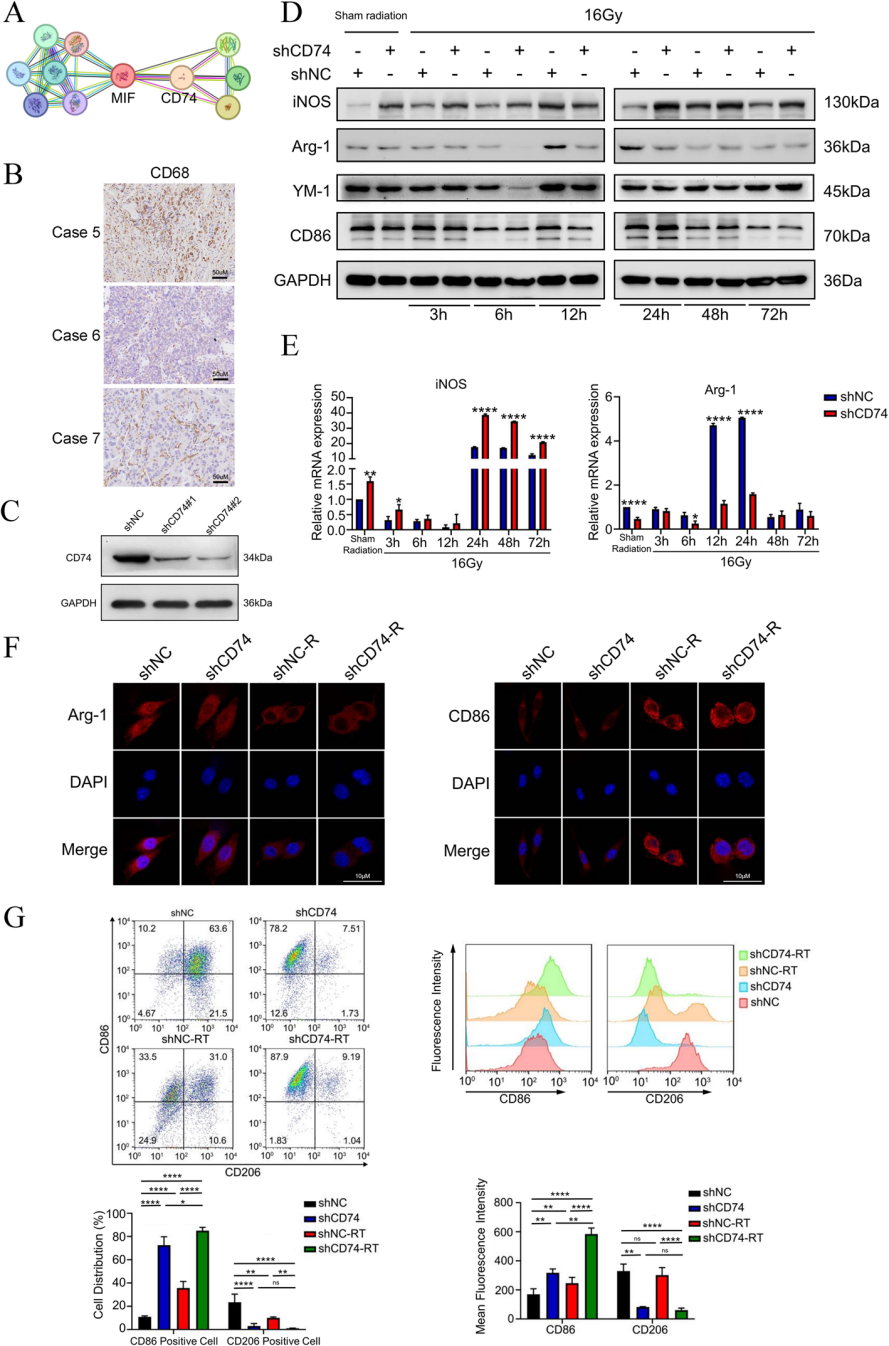
Inhibiting CD74 in microglia in combination with radiation synergistically promoted M1 polarization **a**. Molecules interacting with MIF in STRING database. **b**. IHC staining of CD86 in NSCLC brain metastatic tissue. **c**. Western blotting of CD74 in BV2 cells transduced with either shNC or shCD74 Lentivirus. **d**. Western blotting for the expression of M1 marker iNOS, CD86 and M2 marker Ym-1, Arg-1 in shNC-BV2 cell or shCD74-BV2 cell in sham radiation and 3, 6, 12, 24, 48, and 72 h after 16-Gy radiation. **e**. Total RNA of shNC-BV2 cell or shCD74-BV2 cell was extracted in sham radiation and 3, 6, 12, 24, 48, and 72 h after radiation, and mRNA level of iNOS and Arg-1 were analyzed by real-time qPCR (*n* = 3). **f**. Representative microphotographs of immunofluorescence staining showing expression of Arg-1(left) and CD86(right) in shNC-BV2 cell or shCD74-BV2 cell with or without 16 Gy radiation at 6 h after radiation. **g**. The ratio of CD86 positive and CD206 positive BV-2 cell was analyzed by flow cytometry and the data was processed with FlowJo (version 10.0) program (left). The mean fluorescence intensity of CD86 and CD206 in BV-2 cell was analyzed by flow cytometry and the data was processed with FlowJo (version 10.0) program (right), *n* = 3

To identify the potential molecular interacting proteins with MIF, we searched the STRING database, which revealed a high correlation between MIF and CD74 (correlation score of 0.986, Fig. 2A). CD74 is a non-polymorphic type II transmembrane glycoprotein, highly expressed in antigen-presenting cells such as macrophages. Besides serving as a molecular chaperone for MHC II molecules, CD74 is a cell membrane high-affinity receptor of MIF, D-dopachrome tautomerase, and bacterial proteins. CD74 can form complexes with CD44 or CXCR2/CXCR4. Upon direct binding of the complex with its ligands, intracellular signaling pathways such as ERK, MAPK, AMPK, and NF-κB can be activated [45]. However, in the context of BM of NSCLC, the impact of radiotherapy on macrophage, particularly on microglia, through the MIF/CD74 axis, remains to be thoroughly investigated. First, we examined expression of CD68 (a macrophage marker) in surgical tumors from brain metastases in NSCLC patients using IHC, which showed macrophage infiltration in brain metastases (Fig. 2B). We speculated that NSCLC-secreted MIF binds to CD74 receptors on the surface of macrophages, thereby inhibiting macrophage function. Then, we investigated the effect of silencing CD74 in BV2 cells on the phenotypic transformation of BV2 cells. We transduction BV-2 cells with CD74 knockdown lentivirus and confirmed the knock-down effect of CD74 in BV2 cells by Western Blot (Fig. 2C). Our previous research has shown that microglia were activated after exposure to high-dose radiation [59]. Consequently, BV2 cells were exposed to a single dose of 16 Gy radiation in this study and samples were collected at various time points to detect changes in levels of M1 and M2 markers to investigate the role of the CD74 in microglia after radiation. By analysis of Western Blot, we found that expression of iNOS (M1 marker) was significantly upregulated in 6 h, 24 h, 48 h, and 72 h, and the expression of Arg-1 (M2 marker) was significantly downregulated in 6 h, 12 h, 24 h under the condition of 16 Gy radiation and the silence of CD74 in microglia.

Although the expression of YM-1 did not show significant changes, CD86 was upregulated only at certain time points (Fig. 2D). However, the results of Western Blot indicate that the combination of radiation and CD74 silencing contributes to synergistically promoting partial M1 polarization of microglia. Additionally, based on the results of Western Blot, we selected Arg-1 and iNOS as markers for the phenotype and function of microglia to performed an RT-PCR experiments. The results of RT-PCR indicated that the expression of iNOS was significantly upregulated at 24 h, 48 h, and 72 h, while the expression of Arg-1 was significantly downregulated at 12 h and 24 h. Fig. 2E). Data from immunofluorescence staining and flow cytometry analysis displayed that silencing CD74 in radiation-activated microglia further upregulated the level of CD86 fluorescence intensity and proportion of CD86 + cells (Fig. 2F, G). Based on these experimental results, we cannot pinpoint the exact time when the phenotype of microglia starts to change after radiotherapy. We speculate that this may due to subtle variations in the experimental environment, as the phenotype of microglia is sensitive to environmental factors. But it indicated that silencing CD74 in microglia in combination with radiation synergistically promoted M1 polarization.

### CD74 inhibition reversed radiation-induced AKT phosphorylation in microglia and promoted the M1 polarization in combination of radiation

**Fig. 3:**
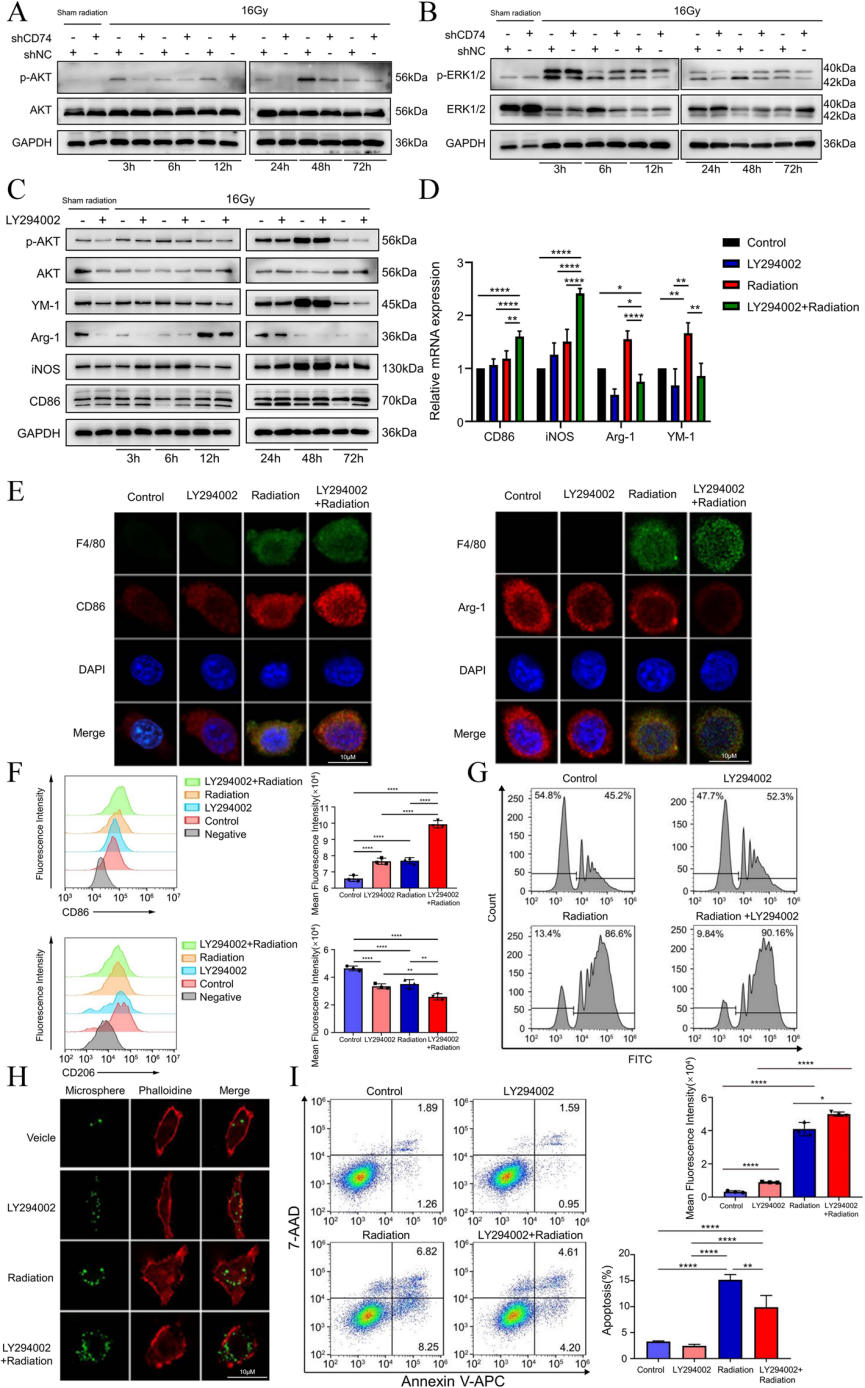
CD74 inhibition reversed radiation-induced AKT phosphorylation in microglia and promoted the M1 polarization in combination of radiation **a**. Western blotting was used for analyzing the change of phosphorylated-AKT(p-AKT) and AKT in shNC-BV2 cell or shCD74-BV2 cell in sham radiation and 3, 6, 12, 24, 48, and 72 h after 16 Gy radiation. **b**. Western blotting for the change of phosphorylated-ERK1/2(p-ERK1/2) and ERK1/2 in shNC-BV2 cell or shCD74-BV2 cell in sham radiation and 3, 6, 12, 24, 48, and 72 h after 16 Gy radiation. **c**. Western blotting for the change of phosphorylated-AKT(p-AKT), AKT, YM-1, Arg-1, iNOS and CD86 in BV2 cell with or without LY294002 in sham radiation and 3, 6, 12, 24, 48, and 72 h after 16-Gy radiation. **d**. Total RNA of BV2 cell was extracted in sham radiation or 16-Gy radiation at 6 h after radiation with or without LY294002, and mRNA level of iNOS, Arg-1, CD86 and YM-1 were analyzed by real-time PCR (*n* = 3). **e**. Representative microphotographs of immunofluorescence staining showing level of F4/80(green)/CD86(red, left) and F4/80(green)/Arg-1(red, right) in BV-2 cultured with or without LY294002 plus 16 Gy radiation at 6 h after radiation. **f**. Mean fluorescence intensity of CD86 and CD206 in BV-2 cell was analyzed by flow cytometry(left) and the data was processed with FlowJo (version 10.0) program (right, *n* = 3). **g**. Mean fluorescence intensity of BV2 cell phagocytic microspheres analyzed by flow cytometry (*n* = 3). **h**. The phagocytic activity of BV2 is defined as the level of green microspheres engulfed by the red phalloidin labelled-BV2. Representative fluorescent images showing phagocytic activity of BV-2 cultured with or without LY294002 plus 16 Gy radiation at 6 h after radiation. **i**. Immunofluorescence was used to analyze the apoptosis rate of BV2 cells (*n* = 3)

Previous studies have found that inhibiting MIF/CD74 signaling pathway can suppress AKT phosphorylation and promote tumor development in melanoma and esophageal squamous cell carcinoma [44, 60]. Besides, the MIF/CD74 signaling inhibits the IFN-γ secretion by promoting ERK1/2 phosphorylation in glioma [61]. Based on these previous studies, we examined the expression of the ERK and AKT signaling pathways in microglia after radiotherapy using Western Blot. As showing in Fig. 3A and B , radiation increased the phosphorylation of AKT, and the AKT phosphorylation was reversed after silencing CD74. However, we did not observe the similar reversion of radiation-induced the phosphorylation of ERK after silening CD74. We also examed the phosphorylation changes of AKT in microglial cells after the addition of MIF recombinant protein using Western Blot. The results showed that MIF recombinant protein could promote the radiation-induced phosphorylation of AKT in microglia. Concurrently, the expression of M1 markers decreased, and the expression of M2 markers increased in microglial after the addition of MIF recombinant protein under radiation conditions (Fig. S1A). In addition, to gauge the changes in microglia phenotype after inhibiting the AKT pathway, we added the AKT inhibitor LY294002 to CD74-silenced BV2 cells. LY294002 penetrates cells and selectively inhibits PI3K, thereby suppressing the PI3K/AKT signaling pathway, including the phosphorylation of AKT [62]. The results showed that after inhibiting p-AKT in irradiated-BV2 cells, M2 markers expression decreased, and M1 markers expression increased, especially at 24 h post-radiation (Fig. 3C). Similarly, RT-qPCR analysis showed that the LY294002 induced the expression of M1 markers and suppressed the expression of M2 markers in irradiated-BV2 cells (Fig. 3D). Immunofluorescence staining analysis also revealed that the LY294002 enhanced CD86 expression and conversely decreased Arg-1 expression (Fig. 3E). Flow cytometry analysis further confirmed that the combination of LY294002 and radiation resulted in a significant increase in fluorescence intensity of CD86 and a decrease in fluorescence intensity of CD206 on BV2 cells (Fig. 3F). In addition, we investigated the effect of LY294002 and radiation on the function of microglia. First, the combination of LY294002 and radiation increased the number of microspheres phagocytosed by BV2 cells (Fig. 3G, H). Apoptotic assay analysis showed that radiation caused the apoptosis of BV2 cells, which were suppressed by LY294002 (Fig. 3I). These findings suggested that inhibiting the CD74-AKT axis supports transformation of microglia into the M1 phenotype after radiation and promotes microglial phagocytosis.

### Blocking the MIF-CD74 interaction between NSCLC and microglia promoted M1 polarization

**Fig. 4:**
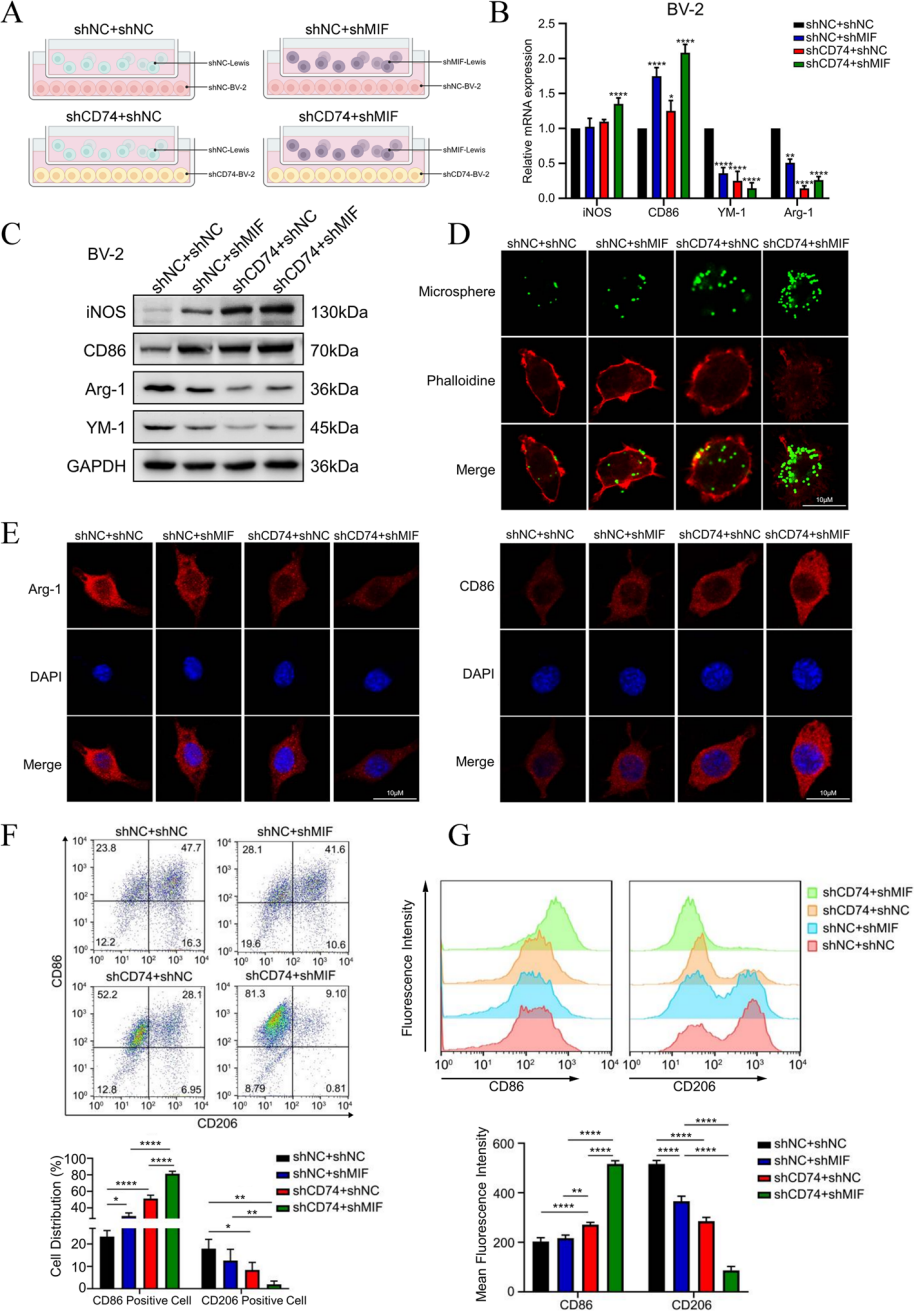
Blocking the MIF-CD74 interaction between NSCLC and microglia promoted microglia M1 polarization **a**. Co-culture sketch map of CD74-shRNA lentivirus transduced BV2 cells and MIF-shRNA lentivirus transduced Lewis cells. After 16-Gy radiation, BV2 cell were immediately co cultured with Lewis cells for 24 h. **b**. Total RNA of BV2 cell was extracted after 24 h co-culture, and mRNA expressions of iNOS, Arg-1, CD86 and YM-1 were analyzed by real-time PCR (*n* = 3). **c**. Western blotting for the expression of iNOS, CD86, Arg-1 and YM-1 in BV2 cell after 24 h co-culture. **d**. Representative fluorescent images showing different phagocytosis of microspheres (green) with phalloidin (red) in BV-2 after 24 h co-culture. **e**. Representative microphotographs of immunofluorescence staining showing expression of Arg-1(left) and CD86(right) in BV-2 cell after 24 h co-culture. **f**. The ratio of CD86 positive and CD206 positive BV-2 cell was analyzed by flow cytometry and the data was processed with FlowJo (version 10.0) program (*n* = 3). **g**. The mean fluorescence intensity of CD86 and CD206 in BV-2 cell was analyzed by flow cytometry and the data was processed with FlowJo (version 10.0) program (*n* = 3)

To the investigate immunomodulatory effect of the MIF/CD74 signaling pathway in BM of NSCLC, we established a co-culture system of Lewis lung cells and BV2 cells to study the effect of substances secreted by Lewis lung cells in the upper chamber on BV2 cells in the lower chamber (Fig. 4A). BV2 cells were co-cultured with Lewis lung cells for 24 h after 16 Gy irradiation, then collect the BV2 cells and detect the expression change of M1 and M2 phenotype markers. Also,we transduction Lewis lung cells with MIF knockdown lentivirus and confirmed the knock-down effect of MIF in Lewis lung cells by Western Blot (Fig. S1E).The data of RT-PCR and Western Blot showed that the CD74-silenced BV2 remarkably increased the expression of M1 markers (iNOS, CD86) after radiation when co-cultured with MIF-silenced Lewis lung cells (Fig. 4B, C). Immunofluorescence showed consistent results (Fig. 4E). Consistently, we confirmed that inhibition MIF in cancer cells and CD74 in microglia promoted M1 polarization, showing upregulated level of CD86 fluorescence intensity and proportion of CD86 + cells (Fig. 4F, G). We further explored how microglia regulated by the MIF/CD74 signaling axis exerts tumor-regulating effects. We hypothesized that the phagocytic capacity of microglia might be altered after phenotypic transformation [63]. The MIF-CD74 blockade between NSCLC and microglia synergistically enhanced microglia phagocytosis after radiation (Fig. 4D). Thus, we suggest that inhibition of Lewis lung cell MIF expression resulted in reduced MIF binding to CD74 receptors on the surface of BV2 cells, which inducing conversion of irradiated-BV2 cells to the M1 phenotype with enhanced phagocytosis.

### Radiation improved tumor hypoxia to decreased HIF-1α dependent MIF secretion by NSCLC

**Fig. 5:**
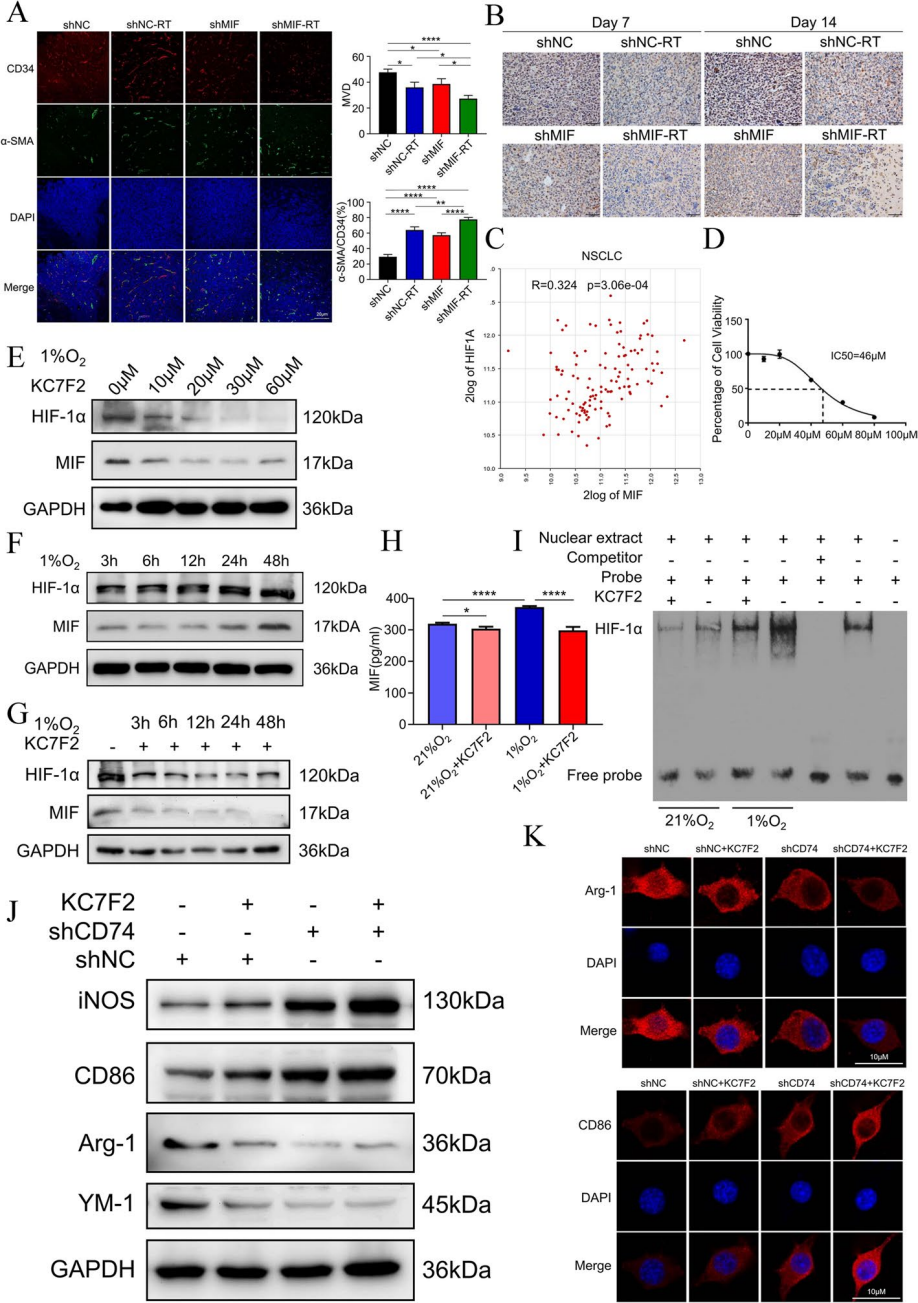
Radiation improved tumor hypoxia and impeded HIF-1α-dependent MIF secretion by NSCLC. **a** Representative microphotographs of immunofluorescence staining showing expression of CD34(red) and α-SMA (green) in shNC or shMIF BM mouse model with or without 10 Gy radiation therapy (*n* = 3). **b**. IHC staining of HIF-1α in brain metastatic tissue of BM mouse model. **c**. Correlation analysis of MIF and HIF-1α in NSCLC patients in R2 database. **d**. Dose–response curves of KC7F2 in Lewis cell. **e**. Western blotting for the level of HIF-1α and MIF in Lewis cell under 1% O_2_ with 0 μM, 10μ, 20 μM, 30 μM, 60 μM KC7F2. **f**. Western blotting for the expression of HIF-1α and MIF in Lewis cell under 1% O_2_ after 3 h, 6 h, 12 h, 24 h, 48 h. **g**. Western blotting for the expression of HIF-1α and MIF in Lewis cell under 1% O_2_ without KC7F2 and with 30 μM KC7F2 after 3 h, 6 h, 12 h, 24 h, 48 h. **h**. Change of MIF concentration in Lewis cell’s supernatant under 21% or 1% O_2_ with or without KC7F2 (*n* = 3). **i**. Binding ability of HIF-1α and MIF promoter in EMSA assay. **j**. Western blotting for the expression of iNOS, CD86, Arg-1 and YM-1 in shNC or shCD74 BV2 cell of the co-culture system. 30 μM KC7F2 was added to the culture medium of Lewis cell in the co-culture system. **k**. Representative microphotographs of immunofluorescence staining showing expression of Arg-1 and CD86 in shNC or shCD74 BV2 cell of the co-culture system. 30 μM KC7F2 was added to the culture medium of Lewis cell in the co-culture system

The effects of radiotherapy and silence of MIF on tumor microvascular structure and HIF-α expression were also examined using the BM model of NSCLC described previously [56]. Pericyte coverage was used to evaluate neovascular maturation and vascular barrier function, with higher pericyte coverage indicating better vascular barrier function. CD34 labeled microvascular endothelial cells and α-SMA labeled pericytes. Tumor tissue micro vessel density (MVD) was significantly lower and pericyte coverage was significantly increased in both the whole-brain irradiation group and MIF-silenced group compared with the control group. The whole-brain irradiation combined with MIF-silenced group had the lowest micro-vessel density and the highest pericyte coverage (Fig. 5A). We also examined the hypoxia within brain metastatic tumor tissues at 7 and 14 days after radiation, as normalization of tumor vasculature further improved tissue hypoxia. Immunohistochemical analysis showed that both whole-brain irradiation and MIF silence resulted in downregulation of HIF-1α expression in brain metastasis, which improved intra-tumor hypoxia (Fig. 5B).

Previous studies have shown that intra-tumor hypoxic microenvironment can induce the release of MIF from tumor cells in breast cancer and HNSCC, leading to tumor growth [64, 65]. We hypothesized that radiation could improve hypoxia in the microenvironment of NSCLC brain metastases, downregulate HIF-1α expression, and cause a decrease in MIF release from tumor cells. By searching the R2 database, we found that HIF-1α expression was significantly and positively correlated with expression of MIF in NSCLC (Fig. 5C,  R = 0.324, *P* < 0.001). The CCK-8 assay data showed that the HIF-1α selective inhibitor KC7F2, which inhibits HIF-1α protein synthesis without affecting its mRNA transcription, at concentrations of 40, 60, and 80 μM significantly inhibited proliferative activity of Lewis cells after 24 h and 48 h of hypoxic culture compared with control group (Fig. S1B). The IC50 concentration of KC7F2 in Lewis cells was 46 μM (Fig. 5D). Expression of HIF-1α was inhibited with the increase of KC7F2 concentration in the hypoxic environment, and expression of HIF-1α was almost the same at 30 μM and 60 μM. Expression of MIF was also inhibited with increase of KC7F2 concentration, which was consistent with the trend of HIF-1α (Fig. 5E), however, the inhibitory effect of KC7F2 on HIF-1α and MIF in Lewis lung cells in a normoxic environment did not change significantly with increasing concentration (Fig. S1C). These results indicated that the inhibitory effect of KC7F2 on HIF-1α and MIF in Lewis cells in a hypoxic environment was concentration-dependent. We determined the concentration of HIF-1α inhibitor KC7F2 to be used at 30 μM. Next, we further explored the incubation time of KC7F2 affecting HIF-1α and MIF expression in Lewis lung cells. The results showed that there was no significant change of HIF-1α and MIF in protein levels within a short period of time (3 h, 6 h). With the prolongation of hypoxia time, the expression of HIF-1α and MIF in Lewis cell increased and reached stability at 24 h and 48 h (Fig. 5F). The Figure 5G showed that the expression of HIF-1α gradually decreased with the prolongation of KC7F2 treating time in the hypoxic environment, reaching stable levels at 12 h and 24 h. The expression of MIF also decreased with the prolongation of treating time, which was basically consistent with the trend of HIF-1α changes. We therefore chose 24 h as the duration of KC7F2 treatment. Lewis cells secreted more MIF under hypoxic conditions when compared with normoxic conditions. Treatment with KC7F2 significantly inhibited MIF secretion (Fig. 5H). Lewis cells were treated with KC7F2 and cultured in a hypoxic (1% O_2_) or normoxic (21% O_2_) incubator for 24 h, and thenthe binding of HIF-1α to the MIF promoter region was examined using the EMSA assay. Hypoxic culture increased the binding of HIF-1α to the MIF promoter compared with normoxic culture (Fig. 5I). The KC7F2 effectively prevented HIF-1α from binding to the MIF promoter.

To investigate how HIF-1α inhibition in cells affects microglia phenotype, we used a co-culture system of Lewis cells and BV2 cells in hypoxic conditions. KC7F2 was added to the conditioned medium, resulting in increased iNOS and CD86 expression and decreased Arg-1 and YM-1 expression in BV2 cells (Fig. 5J). The phenotypic changes were also detected by immunofluorescence staining, revealing increased CD86 expression and decreased Arg-1 expression in activated microglia with the addition of KC7F2 (Fig. 5K). Additionally, adding KC7F2 combined with CD74-silenced in microglia synergistically enhanced microglia phagocytosis after radiation (Fig. S1D).

### MIF inhibition enhanced radiosensitivity for brain metastasis via synergistically promoting microglia M1 polarization in vivo

**Fig. 6:**
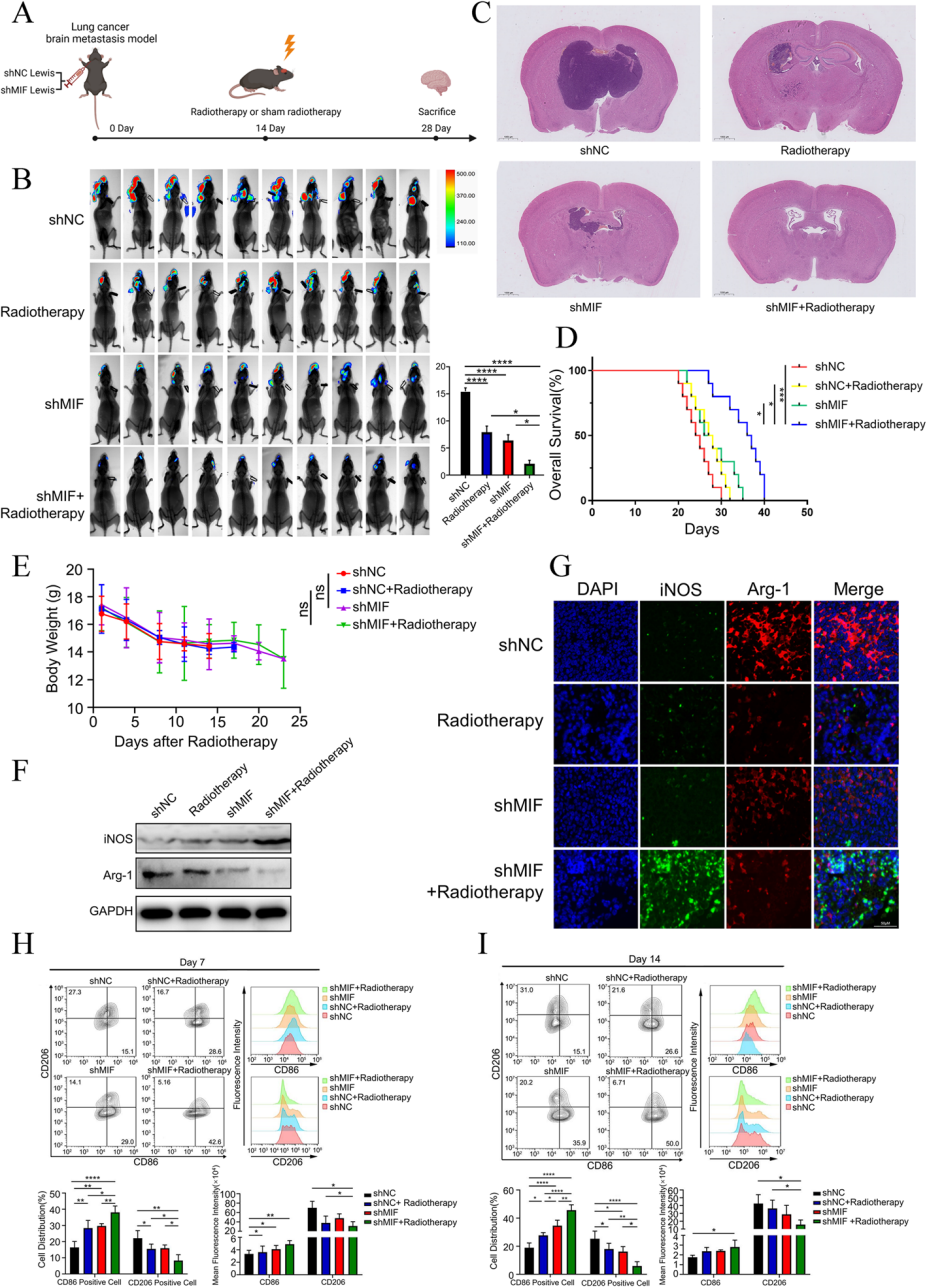
MIF inhibition enhanced radiosensitivity for brain metastasis via synergistically promoting microglia M1 polarization in vivo **a**. The schema for animal studies. **b**. Representative bioluminescent imaging of BM model assay with shNC and shMIF Luc-Lewis cells carotid artery injected in C57BL/6 mice with or without 10 Gy radiotherapy(*n* = 10). **c**. HE staining of BM mice model assay. **d**. Survival analysis for BM mice model (*n* = 5). **e**. Body weight measurement for BM mice model (*n* = 5). **f**. Western blotting for the expression of iNOS and Arg-1 in brain metastasis of BM model. **g**. Immunofluorescence staining of brain metastasis tissue of BM mice model. **h**. At 7 days after radiotherapy, the ratio of CD86 positive and CD206 positive BV-2 cell was analyzed by flow cytometry and the data was processed with FlowJo (version 10.0) program (left). The mean fluorescence intensity of CD86 and CD206 in BV-2 cell was analyzed by flow cytometry and the data was processed with FlowJo (version 10.0) program(right, *n* = 3). **i**. At 14 days after radiotherapy, the ratio of CD86 positive and CD206 positive BV-2 cell was analyzed by flow cytometry and the data was processed with FlowJo (version 10.0) program (left). The mean fluorescence intensity of CD86 and CD206 in BV-2 cell was analyzed by flow cytometry and the data was processed with FlowJo(version 10.0) program(right, *n* = 3)

To further elucidate the effect of MIF in NSCLC cells in vivo, the BM model of mice was established by implanting stably-transduced Luc-Lewis lung cells with MIF-silenced. 14 days after the implant of Lewis lung cells, the formation of BM was confirmed by IVIS, and then, the mice were subjected to a single 10 Gy whole-brain irradiation (Fig. 6A). Analysis of bioluminescence imaging data reveals that silencing MIF in combination with radiotherapy synergistically decrease the fluorescence intensity of BM (Fig. 6B). HE staining showed that tumor cells were distributed in the brain parenchyma, meninges, and ventricles, and in consistent, the volume of BM in combination group was significantly smaller than other groups (Fig. 6C). These results suggest that the silence of MIF in Lewis lung cells could significantly inhibit the growth of BM of NSCLC, and combined with radiotherapy could further enhance its anti-tumor effect. In addition, we found the silencing MIF could significantly prolong the survival time of BM mice, especially in the group combined with radiotherapy (Fig. 6D), and showed no significant difference in body weight between different groups (Fig. 6E). Consistent with experiments in vitro, Western blot and immunofluorescence in vivo showed silencing MIF in Lewis in combination with radiotherapy synergistically increasing levels of M1 phenotype markers, and decreasing levels of M2 phenotype markers (Fig. 6F, G). Flow cytometry analysis further confirmed that the combination of silencing MIF in Lewis and radiotherapy resulted in a significant increase in fluorescence intensity of CD86 and a decrease in fluorescence intensity of CD206 on BV2 cells, no matter it was 7 days or 14 days after Luc-Lewis injection (Fig. 6H, I). The gating strategy was shown in the Fig. S2A. Furthermore, we intraperitoneally injected ISO-1 (MIF inhibitor) in the mouse model of BM. The results revealed that the combined treatment of radiotherapy and ISO-1 significantly reduced the luminescence intensity of BM(Fig. S1F). The results suggest that silencing MIF in combination with radiotherapy synergistically promotes microglia M1 polarization.

### The synergistic antitumor effect of MIF inhibition and radiotherapy was dependent on macrophages

**Fig. 7:**
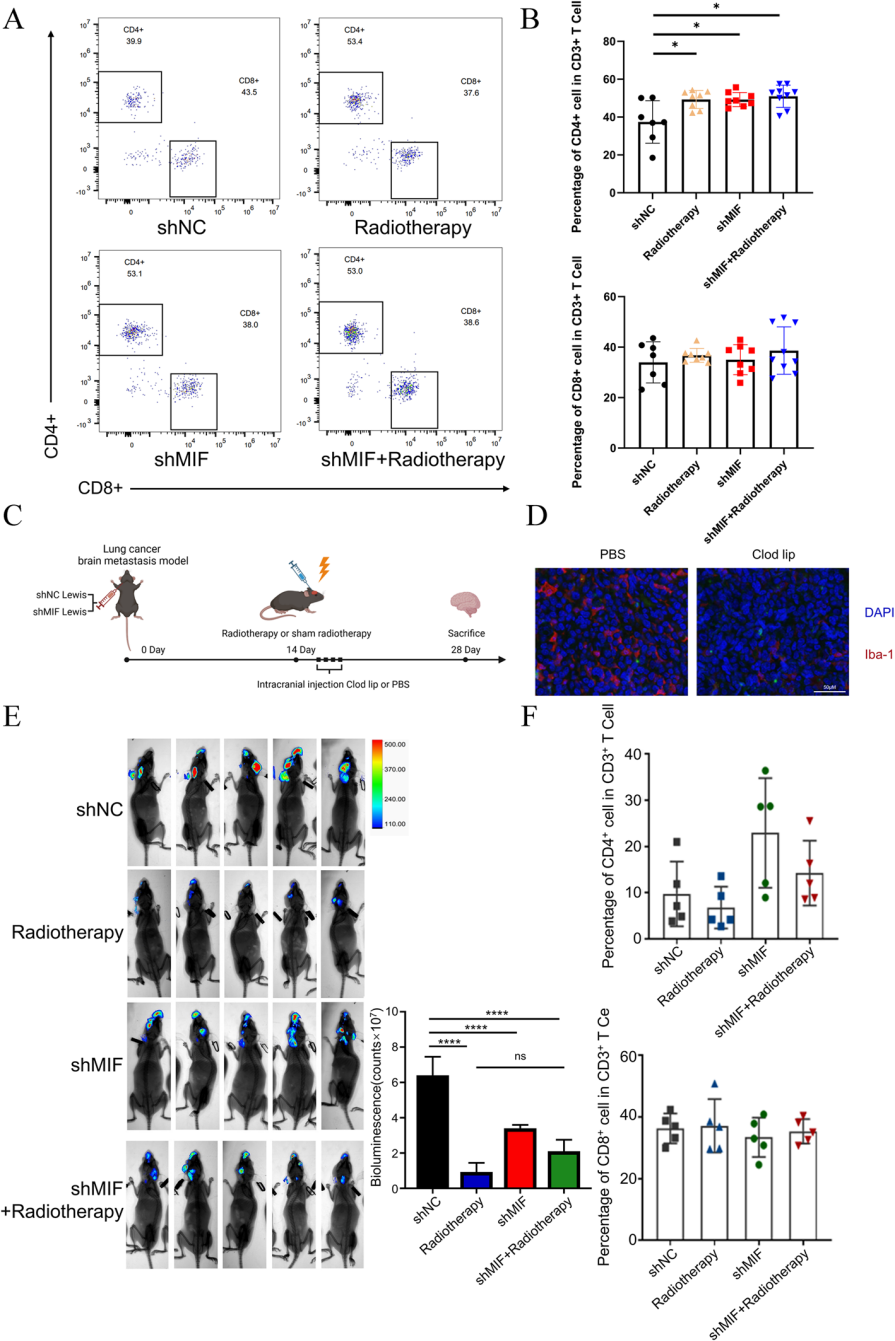
The synergistic antitumor effect of MIF inhibition and radiotherapy was dependent on macrophages **a**. Representative Flowchart in shNC or shMIF brain metastasis tissue of BM mice model. **b** Percentage of CD4^+^ cells in CD3^+^ T cell(left) and percentage of CD8 + cells in CD3 + T cell(right) in shNC or shMIF brain metastasis tissue of BM mice model. **c**. The schema for animal studies. **d**. Immunofluorescence staining of BM mice model with or without Clod lip injection. **e**. Representative bioluminescent imaging of BM model assay with shNC and shMIF Luc-Lewis cells carotid artery injected in C57BL/6 mice with or without 10 Gy radiation therapy after the elimination of microglia (left). Bioluminescence of brain metastasis in BM mice model after treatment as described(right, *n* = 5). **f**. Percentage of CD4^+^ cells in CD3^+^ T cell(left) and percentage of CD8 + cells in CD3 + T cell(right) in shNC or shMIF brain metastasis tissue of BM mice model after the elimination of microglia

We further analyzed whether the proportion of T lymphocytes in the brain tissue of BM mouse model differed between groups. Brains from the four groups were collected and single cell suspensions containing T cells were isolated as described in the method and labelled for different indicators as required using flow cytometry. The gating strategy was shown in the Fig. S2B.The data were processed in FlowJo10 software. As shown in Fig. 7A and 7B, silencing MIF and radiotherapy had an impact on the content of CD4^+^and CD8^+^ T cells in BM, but the results were not statistically significant.

To verify the effect of microglia, BM model with intracranial macrophage clearance was established (Fig. 7C). Immunofluorescence staining of brain tissue showed a significant reduction in macrophage expression in the injected group (Fig. 7D), and the intracranial macrophage clearance model was successfully established. After clearing microglia, we found that the luminescence intensity of the combination group of silencing MIF and radiotherapy was not significantly different from that of the single group, which indicated that the synergistic antitumor effect of silencing MIF and radiotherapy disappeared (Fig. 7E). Flow cytometry was used to observe the change of intra-tumor T cells after intracranial macrophage clearance in four subgroups. As shown in Fig. 7F, there was no significant change in the percentage of CD4+ cells in CD3+ T cells and the percentage of CD8+cells in CD3+ T cells in BM tissue. These results suggest that the synergistic effect of silencing MIF and radiotherapy is dependent on microglia, and has no obvious relationship with T lymphocytes.

## Discussion

BM is one of the major causes of death in advanced NSCLC and is associated with short survival and reduced the quality of life [1]. Radiotherapy is one of the important treatments for BM in advanced NSCLC [3]. However, there are still some patients failed to control the BM after radiotherapy, which are attributed to the occurrence of radiotherapy resistance. The main contribution of this study is to provide NSCLC patients with BM with a potential effective combination therapy strategy, radiotherapy combined with the inhibition of MIF/CD74 axis.

BM of NSCLC possesses a complex tumor microenvironment after radiotherapy, in which macrophages are an important constituent [66]. Macrophages have been shown to exist a pro-tumorigenic effect in a variety of tumors. For example, macrophages at the margin of gliomas secrete cytokines that promote the stemness features of glioma cells, which are closely associated with treatment resistance and recurrence [8]. However, intracranial tumor-associated macrophages are divided into two categories: brain-resident microglia and circulating-derived macrophages [8]. Microglia is an important component of the tumor microenvironment of BM. With different polarization directions, microglia will play opposite roles [67]. However, the factors influencing the phenotype transformation of microglia after radiation are largely unknown. Studies have demonstrated that inhibition of the MIF/CD74 axis in gliomas will promote M1 phenotype polarization of microglia and exert anti-tumor effects [61]. Meanwhile, the phenotype of microglia has an important influence on the efficacy of radiotherapy [68, 69]. Our previous studies have identified the coexistence of M1 and M2 phenotypes in microglia after radiotherapy [59]. However, the role of MIF/CD74 axis in NSCLC patients with BM in radiotherapy is unknown. In this study, we identified MIF as a key molecule in the BM of NSCLC through transcriptomics. We evaluated the effect of blocking MIF/CD74 axis of microglia polarization after radiotherapy. Further, we found that blocking MIF/CD74 axis in vivo could improve the efficacy of radiotherapy and exert anti-tumor effects by inducing microglia polarization toward M1 type after radiotherapy. Inhibition of MIF secreted by tumor cells after radiotherapy could promote M1 polarization of microglia and produce an inhibitory effect on tumor growth.

MIF exhibits very different effects in different diseases due to its complex biological activity. In cancer, MIF/CD74 axis is considered to be a signaling pathway that pro-tumor. Benjamin et al. found that MIF expression is increased in bladder cancer patients, and the use of the MIF inhibitor 4-IPP can suppress tumor growth [70]. Israel et al. discovered that MIF is highly expressed in Luminal B, HER2, and Basal subtypes of breast cancer, but its expression is not prognostically relevant in any subtype of breast cancer [71]. This inconsistency with our findings of MIF expression being associated with the prognosis of NSCLC. We suggest that it may be due to tumor heterogeneity and different tumor microenvironments of breast cancer. Research on the role of the MIF/CD74 axis in the tumor microenvironment of BM of NSCLC is limited. Co-expression of MIF and its putative receptor CD74 in NSCLC is associated with greater tumor vascularity and greater levels of angiogenic CXC chemokines [72]. In colorectal cancer, it was shown that MIF promoted macrophage recruitment and angiogenesis to accelerate tumor progression [73]. Elevated MIF expression supported tumor growth while a loss of MIF promoted the anti-tumor immune infiltration of CD4 + /CD8 + T cells producing IFN in breast cancer [74]. MIF-CD74 interaction directly regulated the expression of PD-L1 and helps tumor cells escape from anti-tumorigenic immune responses in melanoma cells [47]. Previous studies have demonstrated that MIF can interact directly with p53 to stabilize the binding of p53 to its inhibitor mdm2, which in turn leads to a decrease in the expression of p21 and BAX protein and thus disrupts the cell cycle [48]. MIF can activate myeloid-derived suppressor cells (MDSCs) and tumor-associated macrophages (TAM), which act together to inhibit T cells and thus leading to the generation of a microenvironment that promotes tumor growth [75]. Our research found that in the BM of NSCLC, the MIF/CD74 axis primarily influences the efficacy of radiotherapy through modulation of microglia, with minimal association with T cells. We speculate that this is due to the presence of the blood–brain barrier, which makes it difficult for circulating T cells to enter the brain metastases. In addition, MIF can promote tumor cell overexpression of growth mediators and vascular growth factors by activating MAPK signaling pathway [76]. In this study, we first confirmed the high expression of MIF in the BM of NSLCL and BM samples from patients. Secondly, we demonstrated that blocking MIF/CD74 axis can improve the radiotherapy efficacy of BM in NSCLC. These studies provide a new therapeutic target for BM in NSCLC and broaden our understanding of MIF in cancer.

Some study shows that hypoxia mediated transcription of MIF [53]. Other study shows MIF induced by hypoxia in pancreatic adenocarcinoma is necessary for maximal hypoxia-induced HIF1α expression [54]. HIF1α and MIF are interdependence. MIF can regulate and be regulated by HIF1α. HIF1α regulates MIF secretion in AML blasts under hypoxic conditions [77]. In head and neck squamous carcinoma, the HIF-1α–MIF axis contributed to the recruitment of myeloid (CD11b + -Gr-1 +) cells to enhance tumor growth and angiogenesis [65]. Our study demonstrates that, under hypoxic conditions, HIF-1α promotes MIF secretion by increasing its binding to the MIF promoter in NSCLC which is consist with previous study.

In melanoma and esophageal squamous carcinoma, blocking the MIF/CD74 signaling pathway inhibited AKT phosphorylation and promote tumor progression [44, 60], whereas, in glioma, the MIF/CD74 axis inhibited IFN-γ secretion by promoting the phosphorylation of ERK1/2 [61]. In this study, we confirmed that the silence of the MIF/CD74 axis regulates the phenotype Conversion of microglia by inhibiting ATK signaling pathway, rather than ERK1/2, in BM of NSCLC after radiotherapy.

MIF-based therapeutic concepts have been applied to a variety of diseases. A clinical trial (Phase I) of the oxMIF antibody (Imaluma, BAX69) was conducted to investigate the safety, pharmacokinetics (PK), tolerability, and antitumor activity of the antibody in patients with advanced solid tumors [78]. Milatuzumab, a humanized mouse monoclonal to LL1 (anti-CD74) antibody, was evaluated in phase I clinical trial in multiple myeloma [79]. Some small molecule inhibitors, which can inhibit the activity of the MIF/CD74 axis, are also being investigated [80–82]. In this study, we utilized the MIF inhibitor ISO-1 in vivo, demonstrating its inhibitory effect on brain metastases (BM) of NSCLC. It may serve as a potential clinical therapeutic target. Although our study revealed in vitro and in vivo that blockade of the HIF1-α/MIF/CD74 axis by radiation reduced the polarization of microglia to M2-like, generating an immunopermissive niche. Some study showed that MIF inhibition as a strategy for overcoming resistance to immune checkpoint blockade therapy in melanoma [47]. In the future, radiotherapy combining with immune checkpoint inhibitor and MIF inhibitor may have better effect. Further studies are needed to determine whether MIF-based treatment concepts can be combined with radiotherapy in real world in clinic.

## Conclusion

**Fig. 8:**
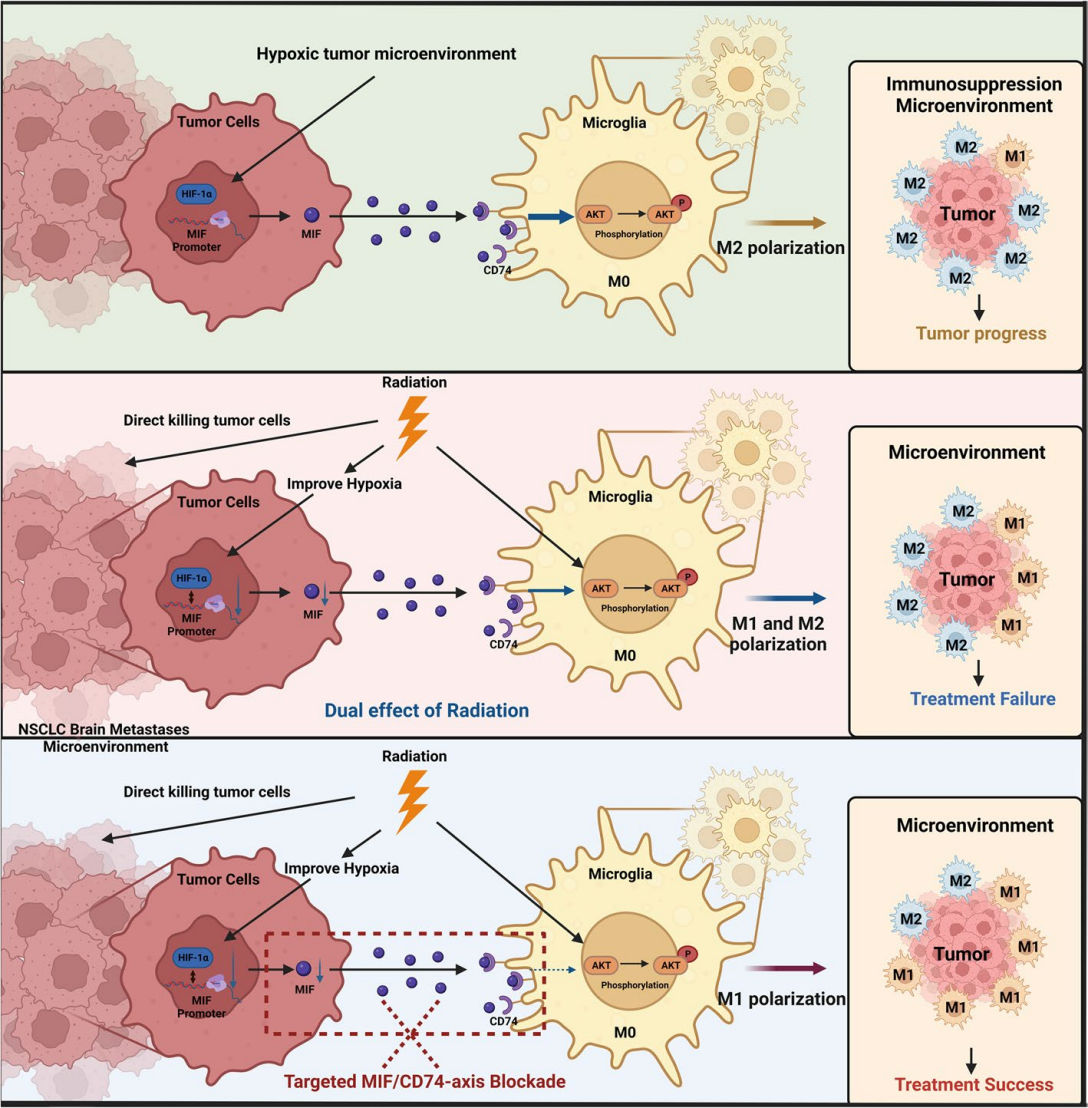
The schematic diagram for blocking the MIF-CD74 axis augments radiotherapy efficacy for brain metastasis in NSCLC via synergistically promoting microglia M1 polarization

In summary, we verified radiation improved the hypoxia of BM of NSCLC, to decrease HIF-1α dependent MIF secretion, and inhibited the binding of MIF to CD74 on microglia. By regulating the phosphorylation of AKT, prompt M1 phenotype, migration and phagocytosis were promoted, thus enhancing the radiotherapy effects. Therefore, the HIF-1α/MIF/CD74 axis could be used as a potential target for radiotherapy, providing a new research basis and theoretical foundation for the treatment of NSCLC brain metastases (Fig. 8).

### Supplementary Information

**Supplementary Material 1.**

**Supplementary Material 2.**

## Data Availability

All data generated or analyzed during this study are included in this published article and its supplementary information files.

## References

[1] Siegel RL, Miller KD, Wagle NS, Jemal A (2023). Cancer statistics, 2023. CA Cancer J Clin.

[2] Waqar SN, Samson PP, Robinson CG, Bradley J, Devarakonda S, Du L (2018). Non-small-cell Lung Cancer With Brain Metastasis at Presentation. Clin Lung Cancer.

[3] Vogelbaum MA, Brown PD, Messersmith H, Brastianos PK, Burri S, Cahill D (2022). Treatment for Brain Metastases: ASCO-SNO-ASTRO Guideline. J Clin Oncol.

[4] Khan IM, Khan SU, Sala HSS, Khan MU, Ud Din MA, Khan S (2023). TME-targeted approaches of brain metastases and its clinical therapeutic evidence. Front Immunol.

[5] Berg TJ, Marques C, Pantazopoulou V, Johansson E, von Stedingk K, Lindgren D (2021). The Irradiated Brain Microenvironment Supports Glioma Stemness and Survival via Astrocyte-Derived Transglutaminase 2. Can Res.

[6] Quail DF, Joyce JA (2017). The Microenvironmental Landscape of Brain Tumors. Cancer Cell.

[7] Jin Y, Kang Y, Wang M, Wu B, Su B, Yin H (2022). Targeting polarized phenotype of microglia via IL6/JAK2/STAT3 signaling to reduce NSCLC brain metastasis. Signal Transduct Target Ther.

[8] Hambardzumyan D, Gutmann DH, Kettenmann H (2016). The role of microglia and macrophages in glioma maintenance and progression. Nat Neurosci.

[9] Foo SL, Sachaphibulkij K, Lee CLY, Yap GLR, Cui J, Arumugam T (2022). Breast cancer metastasis to brain results in recruitment and activation of microglia through annexin-A1/formyl peptide receptor signaling. Breast cancer research : BCR.

[10] Khan F, Pang L, Dunterman M, Lesniak MS, Heimberger AB, Chen P. Macrophages and microglia in glioblastoma: heterogeneity, plasticity, and therapy. The Journal of clinical investigation. 2023;133(1). 10.1172/JCI163446. 10.1172/JCI163446 PMC9797335 36594466

[11] Xuan W, Lesniak MS, James CD, Heimberger AB, Chen P (2021). Context-Dependent Glioblastoma-Macrophage/Microglia Symbiosis and Associated Mechanisms. Trends Immunol.

[12] Cheng N, Bai X, Shu Y, Ahmad O, Shen P (2021). Targeting tumor-associated macrophages as an antitumor strategy. Biochem Pharmacol.

[13] Mantovani A, Sica A, Sozzani S, Allavena P, Vecchi A, Locati M (2004). The chemokine system in diverse forms of macrophage activation and polarization. Trends Immunol.

[14] Shapouri-Moghaddam A, Mohammadian S, Vazini H, Taghadosi M, Esmaeili SA, Mardani F (2018). Macrophage plasticity, polarization, and function in health and disease. J Cell Physiol.

[15] Chen P, Piao X, Bonaldo P (2015). Role of macrophages in Wallerian degeneration and axonal regeneration after peripheral nerve injury. Acta Neuropathol.

[16] Lin Y, Xu J, Lan H (2019). Tumor-associated macrophages in tumor metastasis: biological roles and clinical therapeutic applications. J Hematol Oncol.

[17] Laoui D, Movahedi K, Van Overmeire E, Van den Bossche J, Schouppe E, Mommer C (2011). Tumor-associated macrophages in breast cancer: distinct subsets, distinct functions. Int J Dev Biol.

[18] Mantovani A, Sozzani S, Locati M, Allavena P, Sica A (2002). Macrophage polarization: tumor-associated macrophages as a paradigm for polarized M2 mononuclear phagocytes. Trends Immunol.

[19] Henze AT, Mazzone M (2016). The impact of hypoxia on tumor-associated macrophages. J Clin Investig.

[20] Zhang M, He Y, Sun X, Li Q, Wang W, Zhao A (2014). A high M1/M2 ratio of tumor-associated macrophages is associated with extended survival in ovarian cancer patients. J Ovarian Res.

[21] Zhang H, Li R, Cao Y, Gu Y, Lin C, Liu X (2022). Poor Clinical Outcomes and Immunoevasive Contexture in Intratumoral IL-10-Producing Macrophages Enriched Gastric Cancer Patients. Ann Surg.

[22] Luo P, Lednovich K, Xu K, Nnyamah C, Layden BT, Xu P (2022). Central and peripheral regulations mediated by short-chain fatty acids on energy homeostasis. Transl Res.

[23] Ning J, Ye Y, Bu D, Zhao G, Song T, Liu P (2021). Imbalance of TGF-β1/BMP-7 pathways induced by M2-polarized macrophages promotes hepatocellular carcinoma aggressiveness. Mol Ther.

[24] Jarosz-Biej M, Smolarczyk R, Cichoń T, Kułach N. Tumor Microenvironment as A "Game Changer" in Cancer Radiotherapy. International journal of molecular sciences. 2019;20(13). 10.3390/ijms20133212. 10.3390/ijms20133212 PMC6650939 31261963

[25] Walton EL (2017). Radiotherapy and the tumor microenvironment: The "macro" picture. Biomed J.

[26] Wu Q, Allouch A, Martins I, Modjtahedi N, Deutsch E, Perfettini JL (2017). Macrophage biology plays a central role during ionizing radiation-elicited tumor response. Biomed J.

[27] Gough MJ, Young K, Crittenden M (2013). The impact of the myeloid response to radiation therapy. Clin Dev Immunol.

[28] Pandey R, Shankar BS, Sharma D, Sainis KB (2005). Low dose radiation induced immunomodulation: effect on macrophages and CD8+ T cells. Int J Radiat Biol.

[29] Ibuki Y, Goto R (1997). Enhancement of NO production from resident peritoneal macrophages by in vitro gamma-irradiation and its relationship to reactive oxygen intermediates. Free Rad Biol Med.

[30] Wang SJ, Haffty B. Radiotherapy as a New Player in Immuno-Oncology. Cancers. 2018;10(12). 10.3390/cancers10120515. 10.3390/cancers10120515 PMC6315809 30558196

[31] Tsai CS, Chen FH, Wang CC, Huang HL, Jung SM, Wu CJ (2007). Macrophages from irradiated tumors express higher levels of iNOS, arginase-I and COX-2, and promote tumor growth. Int J Radiat Oncol Biol Phys.

[32] Chiang CS, Fu SY, Wang SC, Yu CF, Chen FH, Lin CM (2012). Irradiation promotes an m2 macrophage phenotype in tumor hypoxia. Front Oncol.

[33] Xu J, Escamilla J, Mok S, David J, Priceman S, West B (2013). CSF1R signaling blockade stanches tumor-infiltrating myeloid cells and improves the efficacy of radiotherapy in prostate cancer. Can Res.

[34] Chen Y, Song Y, Du W, Gong L, Chang H, Zou Z (2019). Tumor-associated macrophages: an accomplice in solid tumor progression. J Biomed Sci.

[35] Shiao SL, Ruffell B, DeNardo DG, Faddegon BA, Park CC, Coussens LM (2015). TH2-Polarized CD4(+) T Cells and Macrophages Limit Efficacy of Radiotherapy. Cancer Immunol Res.

[36] Song L, Sun Q, Zheng H, Zhang Y, Wang Y, Liu S, et al. Roseburia hominis Alleviates Neuroinflammation via Short-Chain Fatty Acids through Histone Deacetylase Inhibition. Molecular nutrition & food research. 2022:e2200164. 10.1002/mnfr.202200164. 10.1002/mnfr.202200164 PMC9787297 35819092

[37] Foster CC, Fleming GF, Karrison TG, Liao CY, Desai AV, Moroney JW (2021). Phase I Study of Stereotactic Body Radiotherapy plus Nivolumab and Urelumab or Cabiralizumab in Advanced Solid Tumors. Clin Cancer Res.

[38] Malfitano AM, Pisanti S, Napolitano F, Di Somma S, Martinelli R, Portella G. Tumor-Associated Macrophage Status in Cancer Treatment. Cancers. 2020;12(7). 10.3390/cancers12071987. 10.3390/cancers12071987 PMC7409350 32708142

[39] Younes AI, Barsoumian HB, Sezen D, Verma V, Patel R, Wasley M (2021). Addition of TLR9 agonist immunotherapy to radiation improves systemic antitumor activity. Transl Oncol.

[40] Kim YH, Gratzinger D, Harrison C, Brody JD, Czerwinski DK, Ai WZ (2012). In situ vaccination against mycosis fungoides by intratumoral injection of a TLR9 agonist combined with radiation: a phase 1/2 study. Blood.

[41] Nobre CC, de Araújo JM, Fernandes TA, Cobucci RN, Lanza DC, Andrade VS (2017). Macrophage Migration Inhibitory Factor (MIF): Biological Activities and Relation with Cancer. Pathol Oncol Res.

[42] Richard V, Kindt N, Decaestecker C, Gabius HJ, Laurent G, Noël JC (2014). Involvement of macrophage migration inhibitory factor and its receptor (CD74) in human breast cancer. Oncol Rep.

[43] Ives A, Le Roy D, Théroude C, Bernhagen J, Roger T, Calandra T (2021). Macrophage migration inhibitory factor promotes the migration of dendritic cells through CD74 and the activation of the Src/PI3K/myosin II pathway. FASEB J.

[44] Fukuda Y, Bustos MA, Cho SN, Roszik J, Ryu S, Lopez VM (2022). Interplay between soluble CD74 and macrophage-migration inhibitory factor drives tumor growth and influences patient survival in melanoma. Cell Death Dis.

[45] Su H, Na N, Zhang X, Zhao Y (2017). The biological function and significance of CD74 in immune diseases. Inflamm Res.

[46] Bozzi F, Mogavero A, Varinelli L, Belfiore A, Manenti G, Caccia C (2017). MIF/CD74 axis is a target for novel therapies in colon carcinomatosis. J Exp Clin Cancer Res.

[47] de Azevedo RA, Shoshan E, Whang S, Markel G, Jaiswal AR, Liu A (2020). MIF inhibition as a strategy for overcoming resistance to immune checkpoint blockade therapy in melanoma. Oncoimmunology.

[48] Fukaya R, Ohta S, Yaguchi T, Matsuzaki Y, Sugihara E, Okano H (2016). MIF Maintains the Tumorigenic Capacity of Brain Tumor-Initiating Cells by Directly Inhibiting p53. Can Res.

[49] Luo Y, Hou WT, Zeng L, Li ZP, Ge W, Yi C (2020). Progress in the study of markers related to glioma prognosis. Eur Rev Med Pharmacol Sci.

[50] Seike T, Fujita K, Yamakawa Y, Kido MA, Takiguchi S, Teramoto N (2011). Interaction between lung cancer cells and astrocytes via specific inflammatory cytokines in the microenvironment of brain metastasis. Clin Exp Metas.

[51] Eguchi R, Wakabayashi I (2020). HDGF enhances VEGF-dependent angiogenesis and FGF-2 is a VEGF-independent angiogenic factor in non-small cell lung cancer. Oncol Rep.

[52] Jeong H, Lee SY, Seo H, Kim BJ. Recombinant Mycobacterium smegmatis delivering a fusion protein of human macrophage migration inhibitory factor (MIF) and IL-7 exerts an anticancer effect by inducing an immune response against MIF in a tumor-bearing mouse model. Journal for immunotherapy of cancer. 2021;9(8). 10.1136/jitc-2021-003180. 10.1136/jitc-2021-003180 PMC8365831 34389619

[53] Simons D, Grieb G, Hristov M, Pallua N, Weber C, Bernhagen J (2011). Hypoxia-induced endothelial secretion of macrophage migration inhibitory factor and role in endothelial progenitor cell recruitment. J Cell Mol Med.

[54] Winner M, Koong AC, Rendon BE, Zundel W, Mitchell RA (2007). Amplification of tumor hypoxic responses by macrophage migration inhibitory factor-dependent hypoxia-inducible factor stabilization. Can Res.

[55] Dewhirst MW, Cao Y, Li CY, Moeller B (2007). Exploring the role of HIF-1 in early angiogenesis and response to radiotherapy. Radiotherapy Oncol.

[56] Wei C, Dong X, Lu H, Tong F, Chen L, Zhang R (2019). LPCAT1 promotes brain metastasis of lung adenocarcinoma by up-regulating PI3K/AKT/MYC pathway. J Exp Clin Cancer Res.

[57] Martin E, El-Behi M, Fontaine B, Delarasse C. Analysis of Microglia and Monocyte-derived Macrophages from the Central Nervous System by Flow Cytometry. Journal of visualized experiments : JoVE. 2017(124). 10.3791/55781. 10.3791/55781 PMC5608497 28671658

[58] Luo Z, Thorvaldson L, Blixt M, Singh K. Determination of Regulatory T Cell Subsets in Murine Thymus, Pancreatic Draining Lymph Node and Spleen Using Flow Cytometry. Journal of visualized experiments : JoVE. 2019(144). 10.3791/58848. 10.3791/58848 30882793

[59] Yang N, Gao X, Qu X, Zhang R, Tong F, Cai Q (2016). PIDD Mediates Radiation-Induced Microglia Activation. Radiat Res.

[60] Liu RM, Sun DN, Jiao YL, Wang P, Zhang J, Wang M (2018). Macrophage migration inhibitory factor promotes tumor aggressiveness of esophageal squamous cell carcinoma via activation of Akt and inactivation of GSK3β. Cancer Lett.

[61] Ghoochani A, Schwarz MA, Yakubov E, Engelhorn T, Doerfler A, Buchfelder M (2016). MIF-CD74 signaling impedes microglial M1 polarization and facilitates brain tumorigenesis. Oncogene.

[62] Wu X, Pu L, Chen W, Zhao Q, Wu G, Li D (2022). LY294002 attenuates inflammatory response in endotoxin-induced uveitis by downregulating JAK3 and inactivating the PI3K/Akt signaling. Immunopharmacol Immunotoxicol.

[63] Yu F, Wang Y, Stetler AR, Leak RK, Hu X, Chen J (2022). Phagocytic microglia and macrophages in brain injury and repair. CNS Neurosci Ther.

[64] Larsen M, Tazzyman S, Lund EL, Junker N, Lewis CE, Kristjansen PE (2008). Hypoxia-induced secretion of macrophage migration-inhibitory factor from MCF-7 breast cancer cells is regulated in a hypoxia-inducible factor-independent manner. Cancer Lett.

[65] Zhu G, Tang Y, Geng N, Zheng M, Jiang J, Li L (2014). HIF-α/MIF and NF-κB/IL-6 axes contribute to the recruitment of CD11b+Gr-1+ myeloid cells in hypoxic microenvironment of HNSCC. Neoplasia (New York, NY).

[66] Wang Y, Chen R, Wa Y, Ding S, Yang Y, Liao J (2022). Tumor Immune Microenvironment and Immunotherapy in Brain Metastasis From Non-Small Cell Lung Cancer. Front Immunol.

[67] Orihuela R, McPherson CA, Harry GJ (2016). Microglial M1/M2 polarization and metabolic states. Br J Pharmacol.

[68] Akkari L, Bowman RL, Tessier J, Klemm F, Handgraaf SM, de Groot M, et al. Dynamic changes in glioma macrophage populations after radiotherapy reveal CSF-1R inhibition as a strategy to overcome resistance. Science translational medicine. 2020;12(552). 10.1126/scitranslmed.aaw7843. 10.1126/scitranslmed.aaw7843 32669424

[69] Hajji N, Garcia-Revilla J, Soto MS, Perryman R, Symington J, Quarles CC, et al. Arginine deprivation alters microglial polarity and synergizes with radiation to eradicate non-arginine-auxotrophic glioblastoma tumors. The Journal of clinical investigation. 2022;132(6). 10.1172/JCI142137. 10.1172/JCI142137 PMC8920336 35113813

[70] Woolbright BL, Rajendran G, Abbott E, Martin A, Amalraj S, Dennis K (2023). Role of MIF1/MIF2/CD74 interactions in bladder cancer. J Pathol.

[71] Cotzomi-Ortega I, Nieto-Yañez O, Juárez-Avelar I, Rojas-Sanchez G, Montes-Alvarado JB, Reyes-Leyva J (2021). Autophagy inhibition in breast cancer cells induces ROS-mediated MIF expression and M1 macrophage polarization. Cell Signal.

[72] McClelland M, Zhao L, Carskadon S, Arenberg D (2009). Expression of CD74, the receptor for macrophage migration inhibitory factor, in non-small cell lung cancer. Am J Pathol.

[73] Klemke L, De Oliveira T, Witt D, Winkler N, Bohnenberger H, Bucala R (2021). Hsp90-stabilized MIF supports tumor progression via macrophage recruitment and angiogenesis in colorectal cancer. Cell Death Dis.

[74] Balogh KN, Templeton DJ, Cross JV (2018). Macrophage Migration Inhibitory Factor protects cancer cells from immunogenic cell death and impairs anti-tumor immune responses. PLoS ONE.

[75] Yaddanapudi K, Putty K, Rendon BE, Lamont GJ, Faughn JD, Satoskar A (2013). Control of tumor-associated macrophage alternative activation by macrophage migration inhibitory factor. J Immunol (Baltimore, Md : 1950)..

[76] Zhang J, Zhang G, Yang S, Qiao J, Li T, Yang S (2016). Macrophage migration inhibitory factor regulating the expression of VEGF-C through MAPK signal pathways in breast cancer MCF-7 cell. World J Surg Oncol.

[77] Abdul-Aziz AM, Shafat MS, Sun Y, Marlein CR, Piddock RE, Robinson SD (2018). HIF1α drives chemokine factor pro-tumoral signaling pathways in acute myeloid leukemia. Oncogene.

[78] Thiele M, Donnelly SC, Mitchell RA. OxMIF: a druggable isoform of macrophage migration inhibitory factor in cancer and inflammatory diseases. Journal for immunotherapy of cancer. 2022;10(9). 10.1136/jitc-2022-005475. 10.1136/jitc-2022-005475 PMC9528626 36180072

[79] Kaufman JL, Niesvizky R, Stadtmauer EA, Chanan-Khan A, Siegel D, Horne H (2013). Phase I, multicentre, dose-escalation trial of monotherapy with milatuzumab (humanized anti-CD74 monoclonal antibody) in relapsed or refractory multiple myeloma. Br J Haematol.

[80] Xu L, Li Y, Sun H, Zhen X, Qiao C, Tian S (2013). Current developments of macrophage migration inhibitory factor (MIF) inhibitors. Drug Discovery Today.

[81] Orita M, Yamamoto S, Katayama N, Fujita S (2002). Macrophage migration inhibitory factor and the discovery of tautomerase inhibitors. Curr Pharm Des.

[82] O'Reilly C, Doroudian M, Mawhinney L, Donnelly SC (2016). Targeting MIF in cancer: therapeutic strategies, current developments, and future opportunities. Med Res Rev.

