# Supplementary figures and images for: Blocking the MIF-CD74 axis augments radiotherapy efficacy for brain metastasis in NSCLC via synergistically promoting microglia M1 polarization
Source: J Exp Clin Cancer Res. 2024 Apr 29;43:128. doi: 10.1186/s13046-024-03024-9 (PMC11059744; doi:10.1186/s13046-024-03024-9)

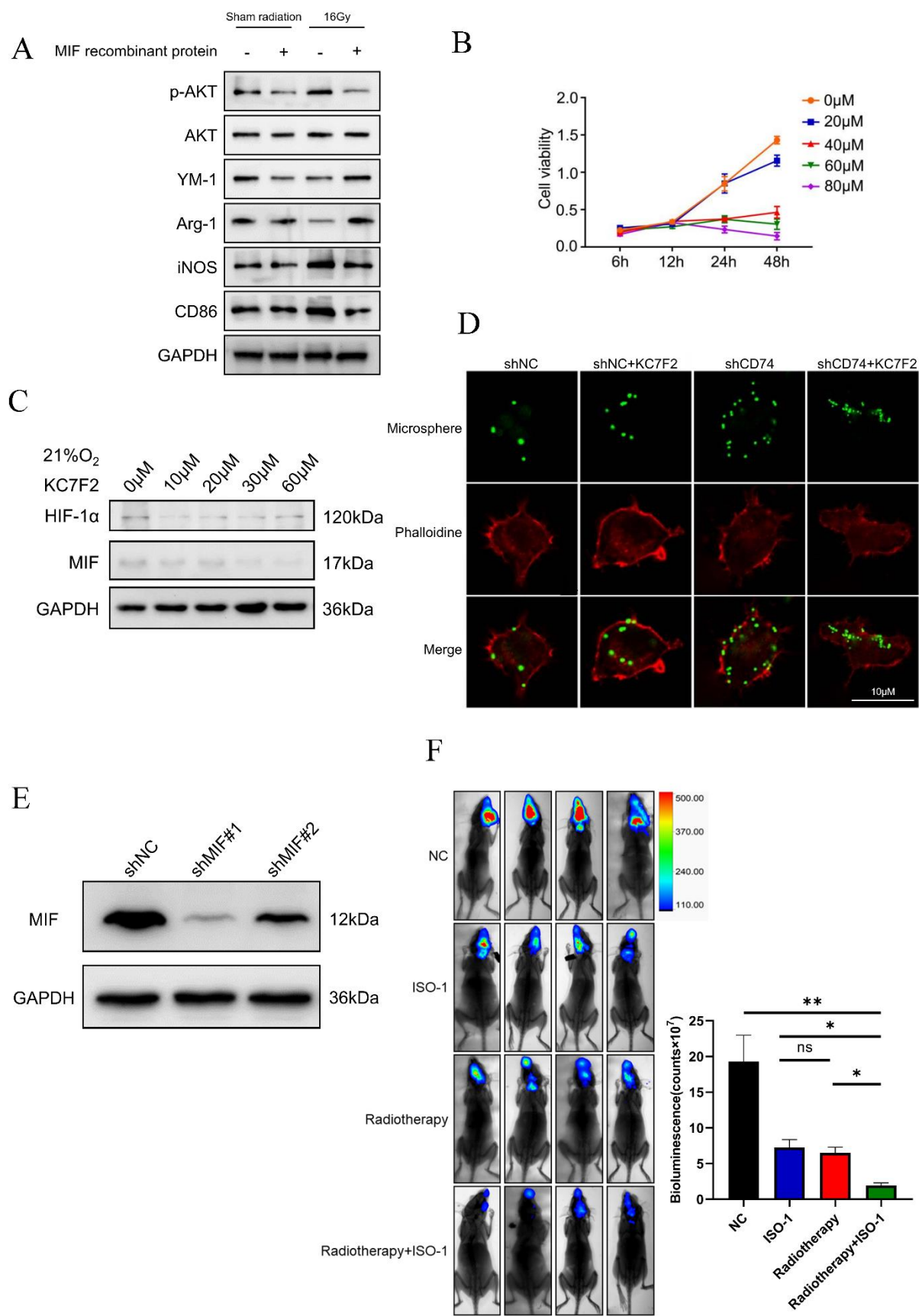

Fig.S1

Supplement: Supplementary file 1 — Supplementary Material 1. [file 13046_2024_3024_MOESM1_ESM.pdf]

A

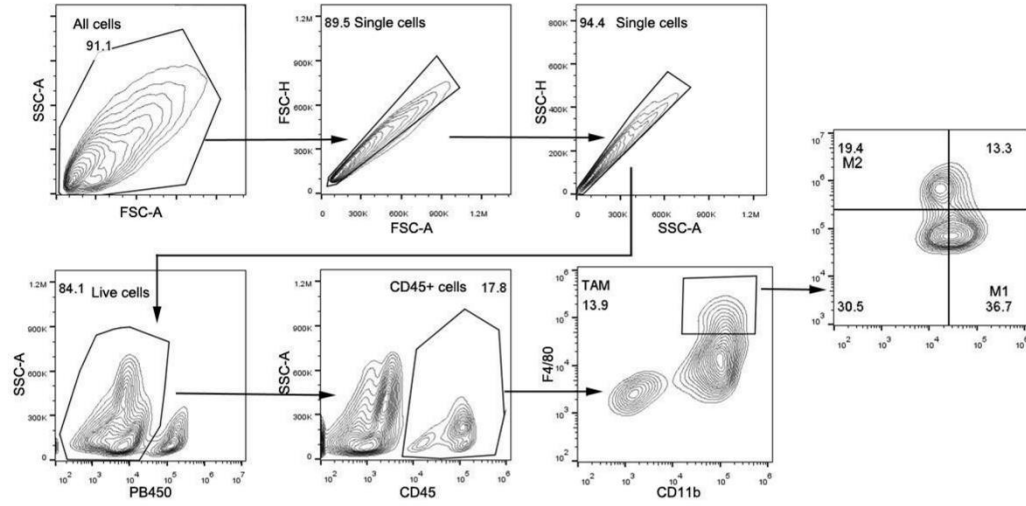

B

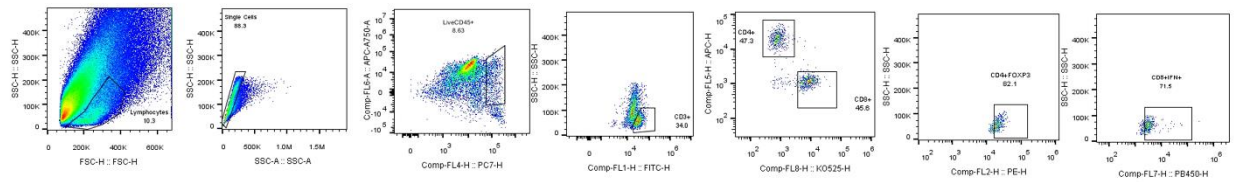

Fig.S2

Supplement: Supplementary file 2 — Supplementary Material 2. [file 13046_2024_3024_MOESM2_ESM.pdf]
